# Adsorptive Molecular Sieving of Styrene over Ethylbenzene by Trianglimine Crystals

**DOI:** 10.1021/jacs.0c13019

**Published:** 2021-03-10

**Authors:** Avishek Dey, Santanu Chand, Bholanath Maity, Prashant M. Bhatt, Munmun Ghosh, Luigi Cavallo, Mohamed Eddaoudi, Niveen M. Khashab

**Affiliations:** † Smart Hybrid Materials (SHMs) Laboratory, Advanced Membranes and Porous Materials Center, King Abdullah University of Science and Technology (KAUST), Thuwal 23955-6900, Kingdom of Saudi Arabia; ‡ King Abdullah University of Science and Technology (KAUST), KAUST Catalysis Center (KCC), Thuwal 23955-6900, Kingdom of Saudi Arabia; § Functional Materials Design, Discovery and Development Research Group, Advanced Membranes and Porous Materials Center, Division of Physical Sciences and Engineering, King Abdullah University of Science and Technology (KAUST) Thuwal 23955-6900, Kingdom of Saudi Arabia

## Abstract

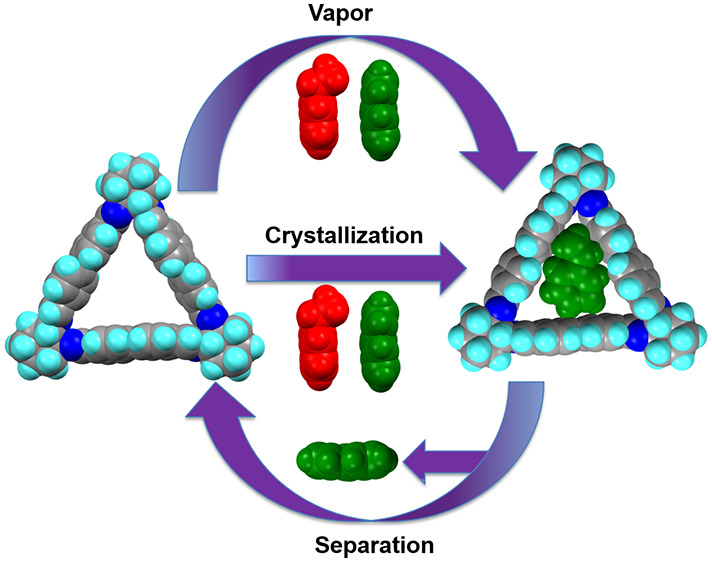

The separation of styrene (ST) and ethylbenzene (EB) mixtures is of great importance in the petrochemical and plastics industries. Current technology employs multiple cycles of energy-intensive distillation due to the very close boiling points of ST and EB. Here, we show that the molecular sieving properties of easily scalable and stable trianglimine crystals offer ultrahigh selectivity (99%) for styrene separation. The unique molecular sieving properties of trianglimine crystals are corroborated by DFT calculations, suggesting that the incorporation of the nonplanar EB requires a significant deformation of the macrocyclic cavity whereas the planar ST can be easily accommodated in the cavity.

Polystyrene is a versatile plastic that is utilized in a wide range of industries ranging from food packaging material to computers and televisions. The global polystyrene market was estimated at $28.5 billion in 2019, making the styrene monomer (ST) one of the most produced aromatic feedstocks in the market.^1^ ST is mainly prepared by the catalytic dehydrogenation of ethylbenzene (EB), in which a large fraction (20–40%) of unreacted EB needs to be removed in order to attain the requisite pure styrene product. The close physical properties of ST and EB necessitate the present use of an energy-intensive separation process based on vacuum and extractive distillation with ∼80 trays and at least four distillation towers to obtain polymer-grade ST (>99.5%).^2,3^ Evidently, the quest for an alternative energy-efficient approach for this important separation is of practical and valuable importance. Notably, adsorption-based separations employing porous materials have achieved excellent performance for hydrocarbon separations through the molecular sieving effect.^4^ Porous zeolites, metal–organic frameworks (MOFs), and covalent organic frameworks (COFs) have facilitated energy-efficient separations of important aromatic and aliphatic petrochemicals.^5−14^ Two MOFs with similar structures, MIL-47 and MIL-53, as well as a flexible Zn based DynaMOF-100, have been successfully used for ST/EB adsorptive separation.^15,16^ More recently, molecular sieving using Cu based MAF-41 afforded ultrahigh selectivity separation of ST with 99.9%+ purity.^17^ However, tuning the porosity of zeolites for selective molecular sieving is quite challenging, while the long-term stability and the liability of the coordination bond have limited the process engineering and industrial transition of some highly selective MOFs.^18^ Lately, nonporous adaptive crystals (NACs) and organic cages have been proven to be extremely stable and versatile for a wide range of molecular sieving applications, including the selective adsorption of ST over EB employing pillar[6]arene.^19−26^ Nonetheless, pillar[6]arene crystals can only work via vapor-based adsorption as they are soluble in both EB and ST.

In this work, an efficient molecular sieving system based on trianglimine host macrocycle **1** is presented for the selective encapsulation and separation of ST from an equimolar ST/EB mixture in both liquid and vapor phase (Figure 1 and Scheme S1). The adsorptive separation selectivity stems from the intrinsic porosity of the host macrocycle that can preferentially accommodate planar ST over nonplanar EB. To the best of our knowledge, this is the first example of using intrinsically porous Schiff base macrocycles for the selective molecular sieving of ST in both solution and vapor phase.

**Figure 1:**
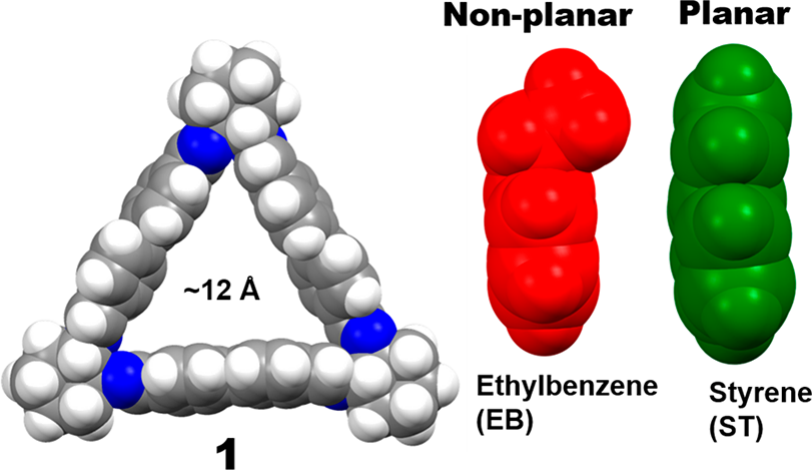
Representation of the crystalline trianglimine host macrocycle **1** and ST and EB guest molecules. Color code: C, gray; N, blue; H, white; EB, red; ST, green.

Macrocycle **1** was prepared following literature reports, as verified by ^1^H NMR (Figure S1), with an intrinsic pore of 12 Å to support ST/EB separation (Figure 1).^27−30^ Single crystal X-ray diffraction (SCXRD) of **1** revealed that two trianglimines are nearly perpendicular to each other, and they are packed as a layered structure with interconnecting channels (Figure S2). Trianglimine **1** did not adsorb N_2_ at 77 K but adsorbed CO_2_ at 195 K (uptake of 70 cm^3^·g^–1^). This is mainly due to the inability of the less polar N_2_ to induce the requisite phase transition compared to CO_2_ and evidenced by the large hysteresis in the CO_2_ adsorption–desorption isotherm (Figure S3). Consequently, crystalline trianglimine **1** can act as a host for a range of relatively small guest molecules such as EB and ST (Figure 1 and Figure S4).

To this end, trianglimine host **1** was crystallized via solution growth method and was investigated by SCXRD with ST and EB guest molecules. Attempts to crystallize **1** in dichloromethane (DCM)/ethyl acetate (EA)/EB (2:1:1 v/v) under slow evaporation resulted in the formation of **EB@1** (Table S1). SCXRD analysis revealed that **EB@1** crystallizes in the triclinic crystal system with chiral *P*1 space group and the asymmetric unit contains four units of trianglimine and two units of EB (Figure 2a). The EB molecules sit inside the intrinsic cavity of the trianglimine and are stabilized by C–H···π interactions (Figures 2b,c and S5a). The packing diagram exhibits a layered structure along the *a* axis where one-dimensional channels are occupied by EB guest molecules (Figure S5b). The guest accessible volume per unit cell is 1694 Å^3^, which is close to 30.6% of the unit cell volume. The estimated electron count for the two disordered EB solvent molecules (116.1 electrons) was confirmed using PLATON SQUEEZE.^31^ Thermogravimetric analysis (TGA) showed a weight loss of 12.2% up to 200 °C, which corresponds to the loss of one EB molecule per macrocycle (Figure S6).

**Figure 2:**
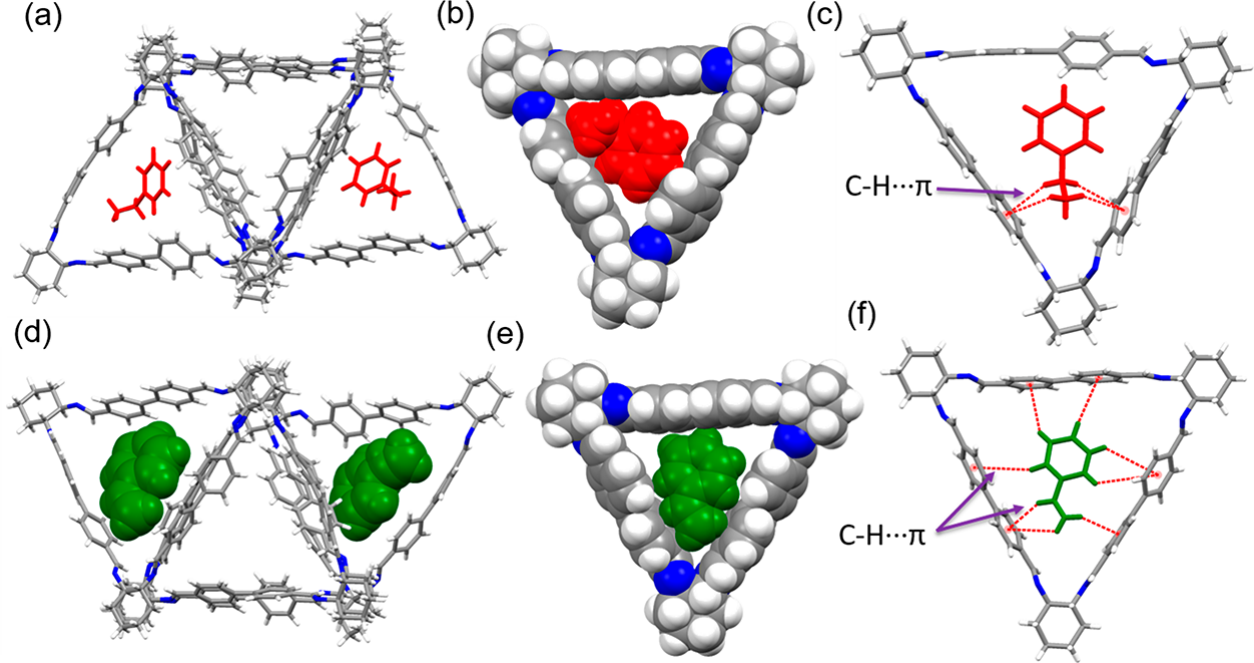
(a) Single crystal structure depicts how EB intercalates with **1** in the asymmetric unit. (b) Space filling structure shows how nonplanar EB fits in the intrinsic cavity of **1**. (c) Host–guest complex is stabilized by C–H···π interactions. (d) SCXRD depicts how ST intercalates with **1** in the asymmetric unit. (e) Space filling structure shows how planar ST fits in the intrinsic cavity of **1**. (f) Host–guest complex is stabilized by C–H···π interactions. Color code: C, gray; N, blue; H, white; EB, red; ST, green.

Similarly, the crystallization of **1** in DCM/EA/ST (2:1:1 v/v) under slow evaporation resulted in the formation of **ST@1** (Table S2). Single crystal analysis revealed that it crystallizes in the same triclinic crystal system and chiral space group with similar asymmetric units as **EB@1** (Figure 2d). The guest ST is stabilized by C–H···π interactions between the ST guest and one of the phenyl rings of the biphenyl spacer (Figures 2e,f and S7a). ST was found to be planar in the crystal lattice (Figures 2e and S7a). The packing diagram indicates that it exhibits a layered structure with one-dimensional channels occupied by ST guest molecules (Figure S7b). The guest accessible volume per unit cell is 1711 Å^3^, which is close to 31% of the unit cell volume. The estimated electron count for the three disordered ST solvent molecules (178 electrons) was confirmed using PLATON SQUEEZE.^31^ TGA shows 11.7% weight loss up to 200 °C, which corresponds to the loss of one ST molecule per macrocycle (Figure S8).

This prompted us to study the competitive crystallization of **1** with a 1:1 v/v mixture of EB and ST in DCM/EA (2:1 v/v). Well-shaped crystals were collected after 3–4 days and SCXRD analysis revealed the selective absorption of ST over EB (Figures 3 and S9–S11). The bulk phase purity of all the guest loaded materials was also verified by powder X-ray diffraction (PXRD). The structure of ST loaded host macrocycle was identical to that of **ST@1** (Figure S11), which indicates that this system is very effective in the crystallization-based separation with a purity of 99.9% as verified by GC analysis (Figure S12). We further performed solid–vapor experiments for single components (EB and ST) as well as mixtures containing EB/ST vapor (Figure 3). Trianglimine **1** was activated upon heating at 70 °C under high vacuum (Figure S13a). Similar amounts of ST and EB were adsorbed when activated **1** was exposed to EB/ST vapor as verified by TGA and ^1^H NMR (Figures 4a and S13b,c). The uptake capacity increases over time and reaches saturation at 48 h. However, time-dependent studies clearly show that the uptake of ST (8 h) was much faster than that of EB (24 h). PXRD patterns of EB and ST loaded **1** were different from **1** but in good agreement with the PXRD patterns of **EB@1** and **ST@1**, respectively. Hence, the adsorption of EB and ST by **1** triggers a structural transformation (Figures 4a–c and S14–S16). To examine whether **1** could differentiate a 1:1 mixture of EB and ST, we conducted a time-dependent solid–vapor study for ST/EB mixture (v/v 1:1). The rates of uptake of EB and ST by **1** were initially competitive over the first 2 h. However, the uptake of ST was found to increase exponentially over time. The ST uptake amount was one molecule per macrocycle (Figures 4d and S17).

**Figure 3:**
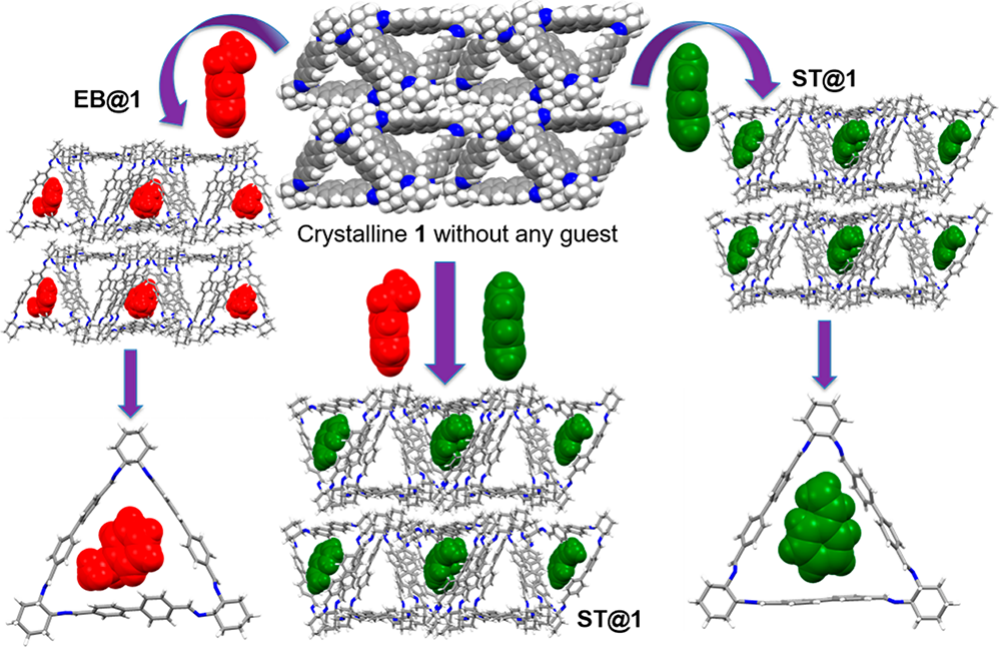
Structural transformation of crystalline host **1** to **EB@1** and **ST@1** for single component adsorption and selectivity toward **ST@1** from the ST/EB mixture in vapor and liquid phase adsorption. Color code: C, gray; N, blue; H, white; EB, red; ST, green.

**Figure 4:**
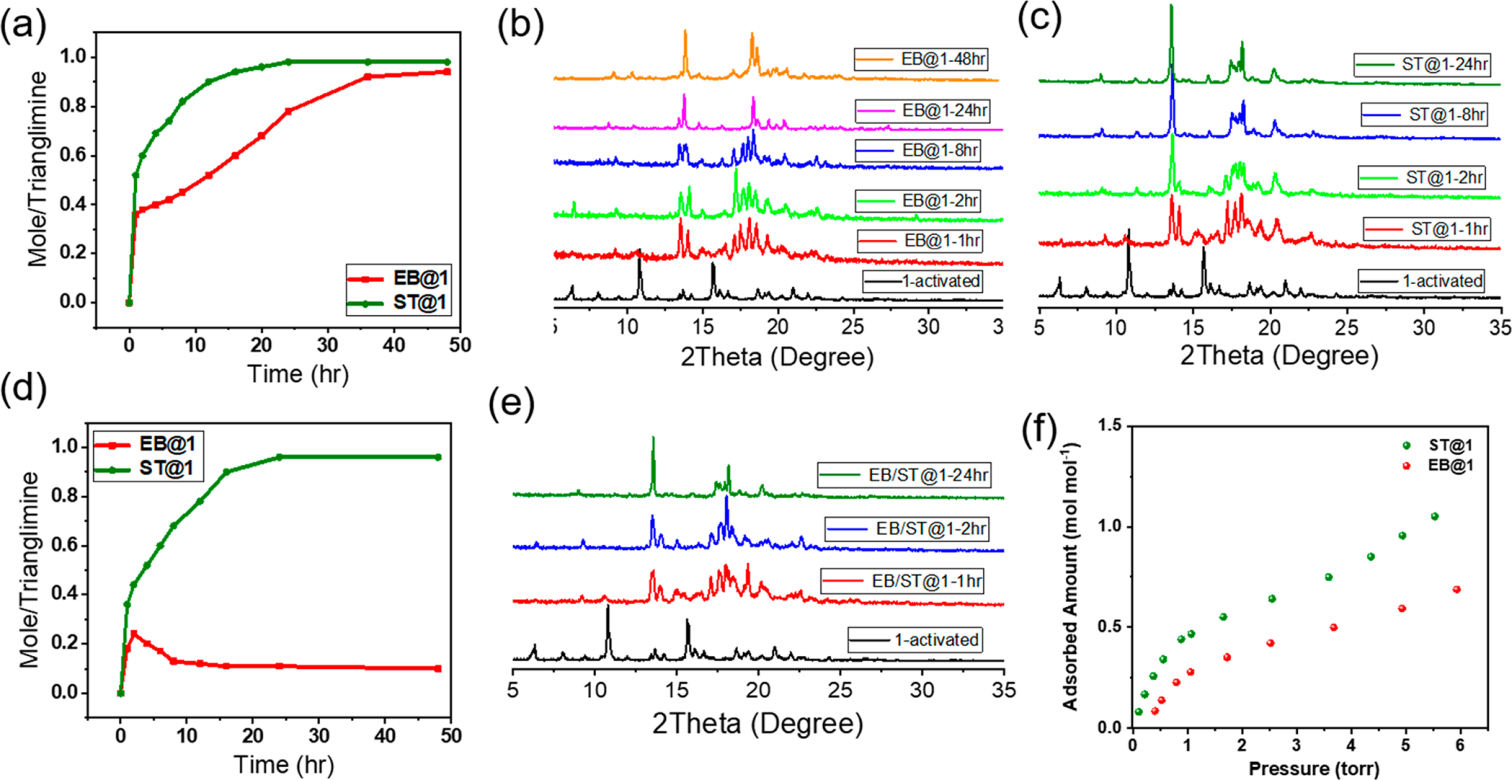
Time-dependent solid–vapor sorption plot. (a) Single component adsorption of activated **1** to form **EB@1** and **ST@1** over time. Time-dependent PXRD of (b) **1** to **EB@1** and (c) **1** to **ST@1**. (d) Selectivity of **ST@1** over **EB@1** in the presence of (1:1 v/v) ST/EB vapor. (e) Time-dependent PXRD of **1** to **ST@1** in the presence of (1:1 v/v) ST/EB vapor. (f) Single component adsorption isotherm of **1** to **EB@1** and **ST@1**.

To gain insight into the structural changes, a time-dependent PXRD experiment was performed to monitor the structural transformation from **1** to **ST@1**. The results show that **1** can selectively capture ST over EB in the pores of the crystalline solid via structural transformation (Figures 4e and S16c). **ST@1** exhibits a *trans*-*trans*-*trans* geometry, while **EB@1** shows *cis*-*trans*-*trans* geometry with respect to the biphenyl group and imine bonds. The “induced fit” of the phenylene rings to optimize the noncovalent interactions appears to be the mode for selectivity toward ST (Figure S18). Overall assembly remains similar with the exception of expansion of discrete voids to guest accessible space with connecting channels (Figure 3). This stipulates the preference towards ST over EB in the vapor phase with 88% purity as confirmed by GC (Figure S19). The slow vapor adsorption lowered the selectivity due to the adsorption of a small quantity of EB on the surface, which can be remediated by heating the materials at 40 °C for 1 h. The recyclability and reversibility was further verified over four cycles without any loss of selectivity (Figures S20 and S21).

To further investigate the adsorption properties of this molecular sieving system, single component vapor adsorption isotherms of EB and ST were measured at 298 K. These results further revealed that the activated samples of crystalline **1** adsorb around 0.69 mol/mol and 1.05 mol/mol of EB and ST, respectively, at around 6 Torr pressure (Figure 4f).

To check the guest loading stability and binding of ST versus EB, we carried out a time-dependent solid vapor experiment using **EB@1** with ST vapor. Over time, EB molecules were gradually replaced by ST, and after 48 h, it reached almost one ST molecule per macrocycle. Time-dependent PXRD patterns further supported the crystalline-to-crystalline phase transformation from **EB@1** to **ST@1** (Figure S22). This supports that **ST@1** is thermodynamically more stable than **EB@1**.

To make it practically more useful given their high separation efficacy, we tested the performance in an 80:20% of ST/EB mixture (2 mL) sample as per the current industrial practices. Interestingly, the host macrocycle **1** transforms to **ST@1** after 24 h as observed in PXRD (Figure S23). After separation, the ratio of ST jumped to 99% compared to 1% for EB as verified by GC analysis (Figure S24). As to stability, the system was stable in water for over 7 days as confirmed by NMR and PXRD (Figure S25).

To further rationalize our findings, we employed density functional theory (DFT) calculations to estimate the thermochemistry of the formation of **EB@1** and **ST@1**. The calculated energy values indicate that the formation of **EB@1** and **ST@1** is thermodynamically feasible by Δ*H*_298_^Sol^ = −31.5 kJ/mol and Δ*G*_298_^Sol^ = −6.4 kJ/mol and Δ*H*_298_^Sol^ = −45.9 kJ/mol and Δ*G*_298_^Sol^ = −19.7 kJ/mol, respectively (Figure S26). In agreement with the experimentally observed selectivity, the formation of **ST@1** was favored by ΔΔ*H*_298_^Sol^ = 14.4 kJ/mol and ΔΔ*G*_298_^Sol^ = 13.3 kJ/mol compared to the formation of **EB@1**.

To gain better insight into the origin of this adsorptive selectivity, distortion-interaction analysis and energy decomposition analysis (EDA) (details in SI, Tables S4 and S5) have been performed on the electronic structures of **EB@1** and **ST@1**. The distortion-interaction analysis shows that the Δ*E*_bond_ of **EB@1** is 14.9 kJ/mol lower than that of **ST@1**, due to the larger contribution of Δ*E*_dis_ of the host fragment in **EB@1**, which is unfavored by 16.6 kJ/mol relative to that of **ST@1** (Table S4). Indeed, a clear distinction is observed between the distorted geometry of **1** in **EB@1** and the optimized geometry of **1**, with a RMSD (root-mean-square deviation) on heavy atoms of 0.59 Å (Figure S27a), while the distorted geometry of **1** in **ST@1** exhibits a closer resemblance to the optimized geometry of **1** (Figure S27b), with a RMSD on heavy atoms of 0.18 Å only. This further supports that the planar ST molecule fits easily inside the cavity of **1**, while incorporation of EB demands a significant deformation of the macrocycle cavity.

In summary, crystalline trianglimine host macrocycles can selectively separate styrene (ST) from ethylbenzene (EB) via adsorptive molecular sieving. The originally nonporous host macrocycle can accommodate both ST and EB as single components; however, it will selectively encapsulate ST from a ST/EB mixture with 99% selectivity. DFT calculations supported our experimental findings that planar ST is a better fit compared to the nonplanar EB. Suggestively, this class of intrinsically porous molecular adsorbents offers great potential to transform traditional separation techniques towards energy-efficient and green industrial practices, capitalizing on the elements of economical and environmental sustainability.

## Supporting Information Available

Detailed synthetic procedures and characterization, including Figures S1–S27 and Tables S1–S5, Scheme S1, NMR spectra, GC, SCXRD data, crystallographic figure and parameters, PXRD, TGA, computational details, and Cartesian coordinates (PDF)

## Accession Codes

CCDC 2061331–2061333 contain the supplementary crystallographic data for this paper. These data can be obtained free of charge via www.ccdc.cam.ac.uk/data_request/cif, or by emailing data_request@ccdc.cam.ac.uk, or by contacting The Cambridge Crystallographic Data Centre, 12 Union Road, Cambridge CB2 1EZ, UK; fax: +44 1223 336033.
