# Supplementary material for: Adsorptive Molecular Sieving of Styrene over Ethylbenzene by Trianglimine Crystals
Source: J Am Chem Soc. 2021 Mar 10;143(11):4090–4. doi: 10.1021/jacs.0c13019 (PMC8041285; doi:10.1021/jacs.0c13019)
Supplement: Supplementary file 1 — ja0c13019_si_001.pdf [file ja0c13019_si_001.pdf]

Supporting Information

Adsorptive Molecular Sieving of Styrene over Ethylbenzene by Trianglimine Crystals

Avishek Dey[1], Santanu Chand[1], Bholanath Maity[2], Prashant M. Bhatt[3], Munmun Ghosh[1], Luigi Cavallo[2], Mohamed Eddaoudi[3], Niveen M. Khashab[1]*

[1]Smart Hybrid Materials (SHMs) Laboratory, Advanced Membranes and Porous Materials Center, King Abdullah University of Science and Technology (KAUST), Thuwal 23955-6900, Kingdom of Saudi Arabia.

[2]King Abdullah University of Science and Technology (KAUST), KAUST Catalysis Center (KCC), Thuwal 23955-6900, Kingdom of Saudi Arabia.

[3]Functional Materials Design, Discovery and Development Research Group, Advanced Membranes and Porous Materials Center, Division of Physical Sciences and Engineering, King Abdullah University of Science and Technology (KAUST) Thuwal 23955-6900, Kingdom of Saudi Arabia.

1. Experimental Section

Materials. All reagents and solvents were purchased from commercial sources and used without further purification. ^{1}H-NMR spectra were performed at 400 MHz with $CDCl_3$ solutions at 5mg/ml concentration with an Avance III Bruker Corporation instrument.

Thermogravimetric Analysis. TGA analysis was carried out using a Q5000 analyzer (TA instruments) with an automated vertical overhead thermobalance. The samples were heated at the rate of 5 °C/min using N_2 as the protective gas.

Powder XRD (PXRD). Powder X-ray diffraction (PXRD) patterns were collected at room temperature on a Bruker D2 Phaser powder diffractometer using Cu Kα radiation (λ = 1.5418 Å, 40 kV and 30 mA). The sample was placed in a zero-background sample holder and normal configuration of the instrument was used.

Gas Chromatography (GC) Analysis. GC measurements were carried out using an Agilent 7890A instrument configured with an FID detector and a DB-624 column (30 m × 0.45 mm × 2.55 μm). The following GC method was used; the oven was programmed from 50°C, ramped at 10°C min^{-1} increments to 150°C with 15 min hold, the total run time was 25 min; injection temperature 250°C; detection temperature 280°C with hydrogen, air, and make-up flow-rates of 35, 300 and 30 mL min^{-1}, respectively; and the helium (carrier gas) flow-rate 3.0 mL min^{-1}. The samples were injected in the split mode (20:1).

Vapor adsorption. Vstar vapor adsorption analyzer from Quantachrome instruments was used for ST and EB adsorption. In a typical experiment, 30 mg sample was activated in-situ at 140°C under dynamic vacuum for 8 hours. The temperature was increased to 140°C from room temperature at a rate of 1°C/min. The activated sample was used for the corresponding isotherm measurement. All the adsorption experiments of organic vapors were carried out at 25°C sample temperature.

N_2 and CO_2 adsorption. N_2 and CO_2 adsorption was carried out using Autosorb-iQ gas adsorption analyzer, (Quantachrome Instruments) equipped with Cryocooler to maintain the temperature at 77 K and 195 K respectively.

Synthesis of Trianglimine 1. A mixture of (1R,2R)-diaminocyclohexane (0.275 g, 2.38 mmol) and 4,4'-diformylbiphenyl (0.5 g, 2.38 mmol) in $CHCl_3$/methanol (1:10) in the presence of triethylamine (2ml) was stirred at room temperature for 48 h. The white precipitate was isolated and washed with MeOH. Crude product was recrystallized from chloroform/ethyl acetate giving macrocycle **1**.

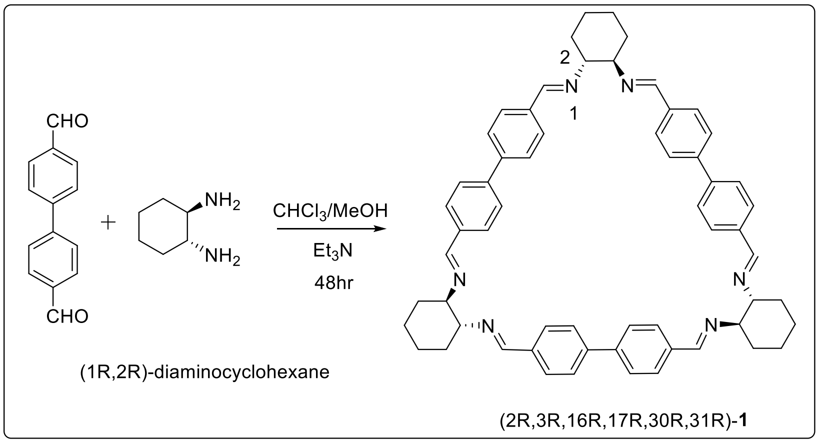

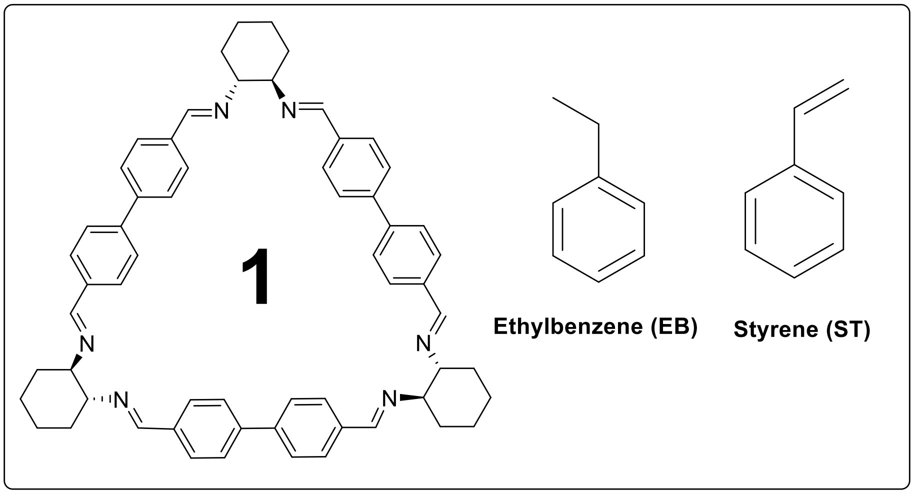

Scheme S1. Chemical drawings of the macrocycle **1**, ethylbenzene (EB) and styrene (ST).

Single Crystal Growth

Crystallization of 1. Macrocycle **1** (10mg, 0.0115 mmol) was taken in a vial and dissolved in (2:1) ml of ethyl acetate (EA)/chloroform ($CHCl_3$). Crystals of **1** were obtained by slow diffusion of hexane in a closed vessel with **1** in EA/$CHCl_3$ for 3 days.

Crystallization of EB@1. 10mg (0.0115 mmol) of macrocycle **1** was dissolved in 2 ml of chloroform ($CHCl_3$). To this, 1 ml ethyl acetate (EA) or 1 ml acetonitrile was added to make system mixed solvent with different polarity. Further, 1 ml ethylbenzene (EB) was added to that solution and kept for crystallization. After 3-4 days block prism shaped single crystals were found and suitable for single crystal X-ray diffraction. Bulk purities was verified by powder X-ray diffraction (PXRD). The crystals of **EB@1** were also obtained by the vapor diffusion of (2:1) ml of EA / $CHCl_3$ solution of **1** in a closed vessel of EB vapor.

Crystallization of ST@1. 10mg (0.0115 mmol) of macrocycle **1** was dissolved in 2 ml of chloroform ($CHCl_3$). To this, 1 ml ethyl acetate (EA) or 1 ml acetonitrile was added to make system mixed solvent with different polarity. Further, 1 ml styrene (ST) was added to that solution and kept for crystallization. After 3-4 days block prism shaped single crystals were found and suitable for single crystal X-ray diffraction. Bulk purities was verified by powder X-ray diffraction (PXRD). The crystals of **ST@1** were also obtained by the vapor diffusion of (2:1) ml of EA / $CHCl_3$ solution of **1** in a closed vessel of ST vapor.

Crystallization of ST@1 from EB/ST. 10mg (0.0115 mmol) of macrocycle **1** was dissolved in 2 ml of chloroform ($CHCl_3$). To this, 1 ml ethyl acetate (EA) or 1 ml acetonitrile was added to make system mixed solvent with different polarity. Further, 1 ml EB and 1 ml of ST was added to that solution and kept for crystallization. After 3 days block prism shaped single crystals were found and suitable for single crystal X-ray diffraction. Bulk purities was verified by powder X-ray diffraction (PXRD).

Vapor-phase adsorption measurements

For each single-component ST and EB adsorption experiment, an open 5 mL vial containing 20mg of activated crystalline materials of **1** (activation was done at 70°C under vacuum) was placed in a sealed 20 mL vial containing 2 mL of ST or EB. The uptake capacity of the crystals of **1** was measured by different time intervals by completely dissolving the crystals in $CDCl_3$ and measuring the ratio of ST or EB by ^{1}H-NMR, respectively. Uptake capacity values were determined from the ratio of each isomer peaks using ^{1}H-NMR and GC following literature protocols.[1]

For two-component ST-EB adsorption experiment, an open 5 mL vial containing 20mg of activated crystalline materials of **1** (activation was done at 70ºC under vacuum) was placed in a sealed 20 mL vial containing mixture of 2 ml of ST/ EB (1:1 v/v). The uptake capacity of the crystals of **1** was measured by different time intervals by completely dissolving the crystals in $CDCl_3$ and measuring the ratio of ST or EB by ^{1}H-NMR, respectively. Selectivity values were determined from the ratio of each isomer peaks using ^{1}H-NMR and GC following literature protocols.[1]

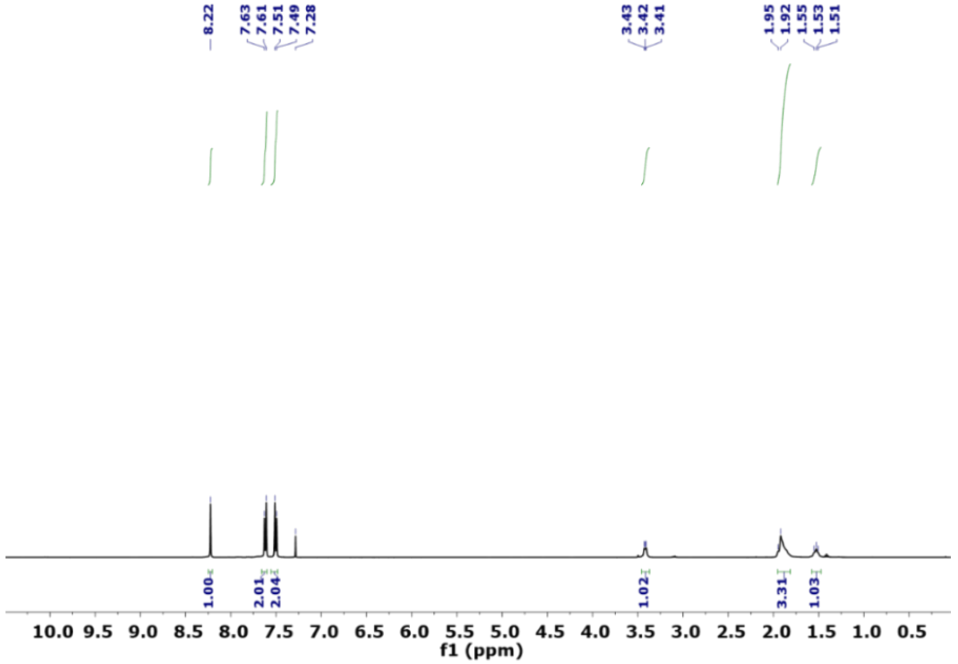

Figure S1. ^{1}H (400 MHz; $CDCl_3$) NMR spectrum of trianglimine **1**. δ 8.22 (1H, s), 7.62 (2H, d, *J*= 8.0 Hz), 7.50 (2H, d, *J*= 8.0 Hz), 3.43-3.41 (1H, m), 1.95-1.92 (3H, m), 1.55-1.51 (1H, m).

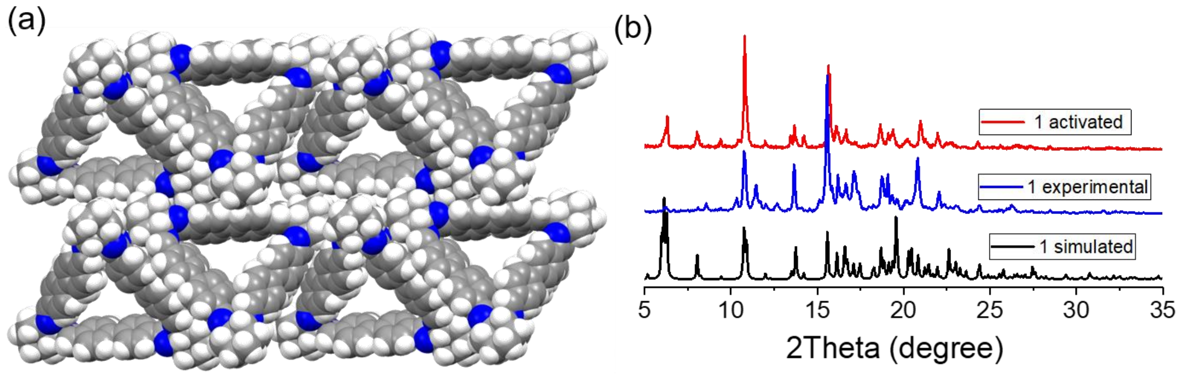

Figure S2. (a) Trianglimines molecules are assembled in a head-tail fashion that generates layered structure with connecting channels (Color code: C, gray; N, blue; H, white). (b) Comparison of experimental, simulated and activated sample PXRD (70°C under vacuum) for **1**.

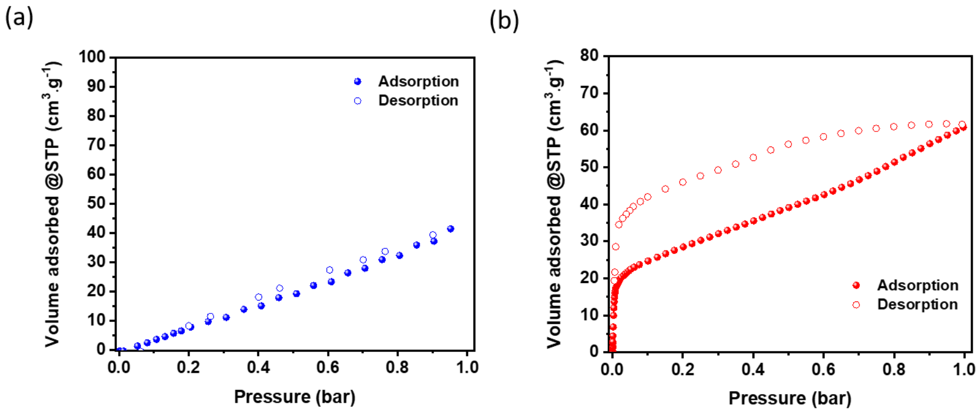

Figure S3. (a) N_2 gas adsorption of **1** at 77K. (b) CO_2 gas adsorption of **1** at 195K.

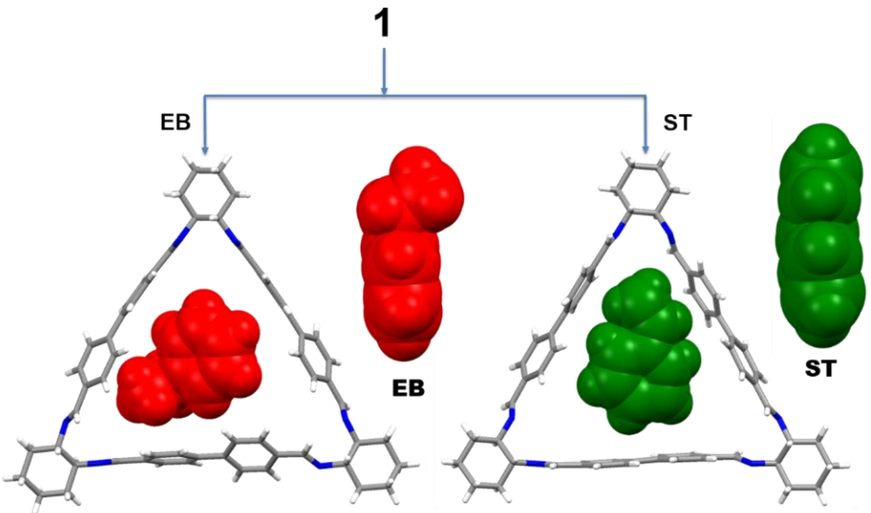

Figure S4. Schematic presentation of the guest EB and ST inclusion by host trianglimine **1** where EB and ST shows nonplanar and planar geometry (Color code: C, gray; N, blue; H, white; EB, red; ST, green).

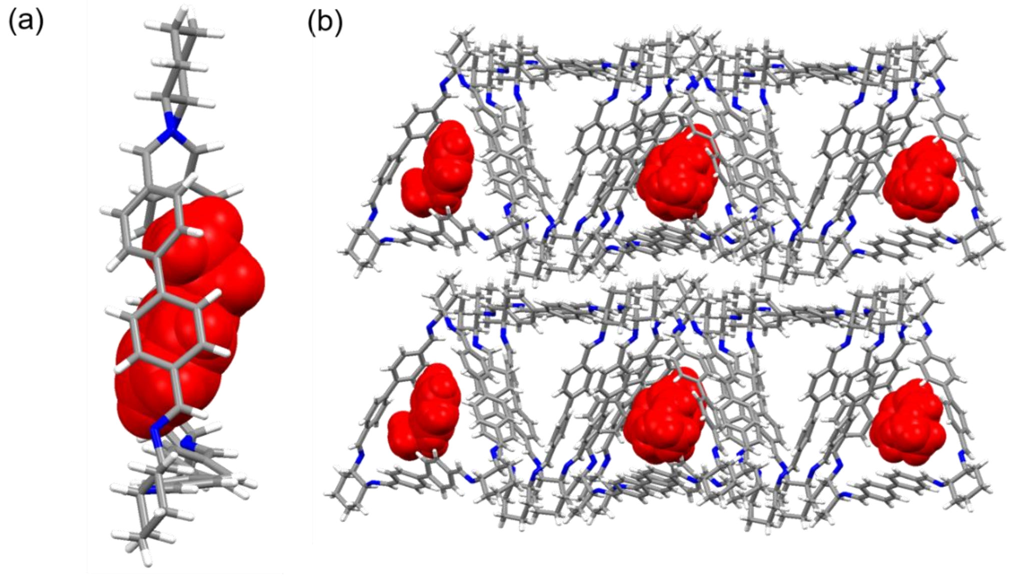

Figure S5. Description of the crystal structure of **EB@1**: (a) Side view shows how non-planar EB fits inside the cavity by the host trianglimine **1**. (b) Packing diagram shows the layered structure with connecting one dimensional triangular channel containing EB (Color code: C, grey; N, blue; H, white; EB, red).

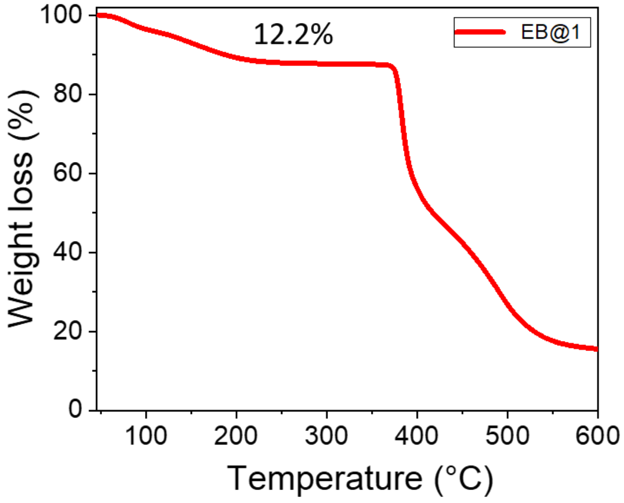

Figure S6. TGA of **EB@1**.

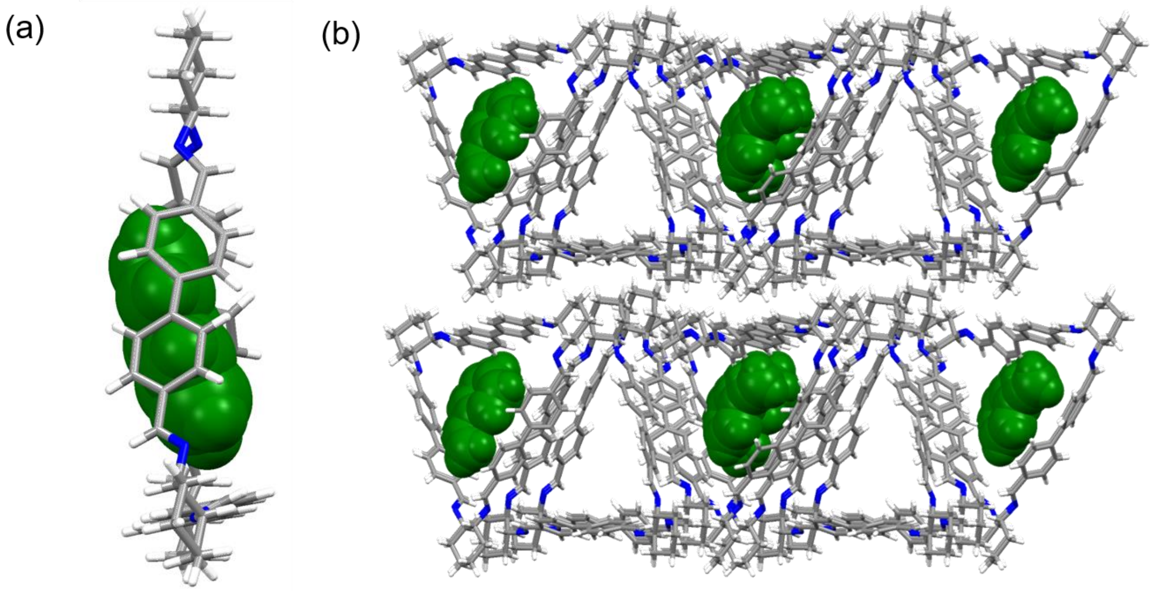

Figure S7. Description of the crystal structure of **ST@1**: (a) Side view shows how planar ST fits in the cavity of the host trianglimine **1**. (b) Packing diagram shows the layered structure with interconnecting one dimensional triangular channels containing ST inside the channels (Color code: C, gray; N, blue; H, white; ST, green).

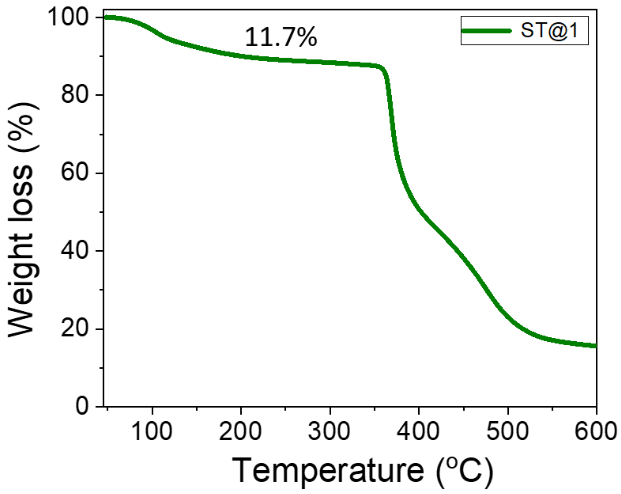

Figure S8. TGA of **ST@1**.

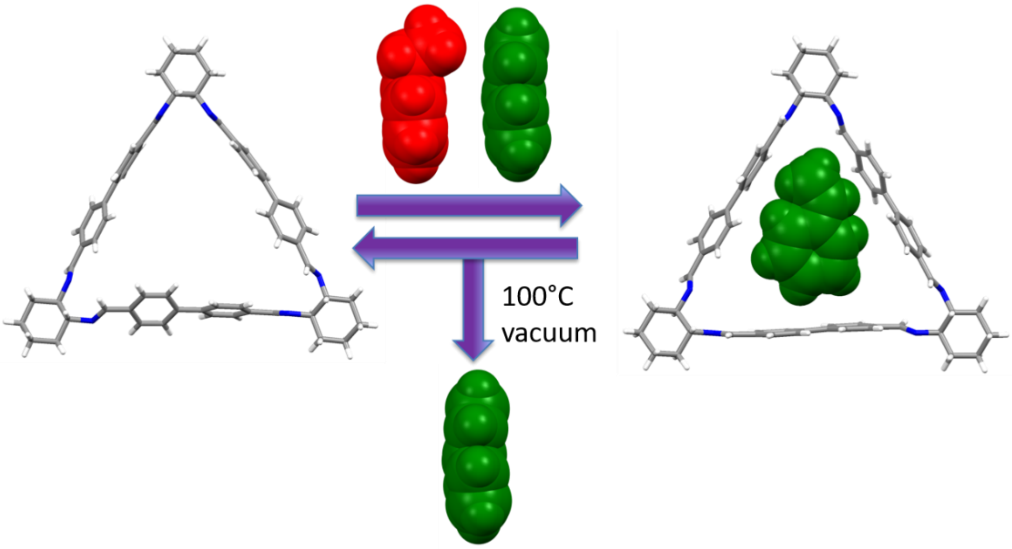

Figure S9. Schematic representation of the selectivity of **1** towards ST in an EB/ST mixture.

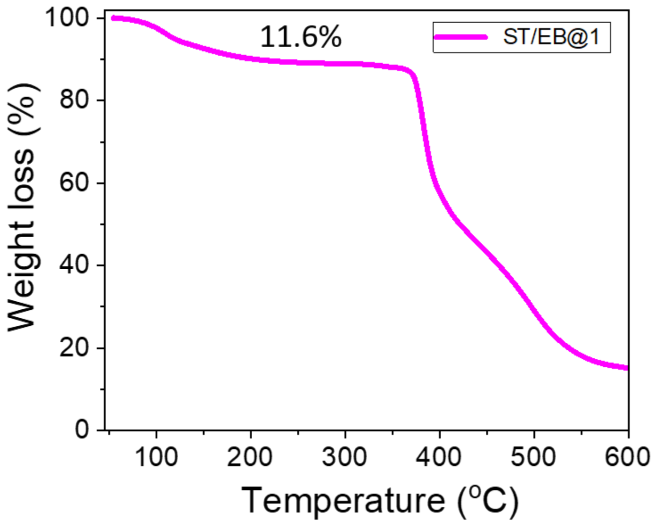

Figure S10. TGA of **EB/ST@1**.

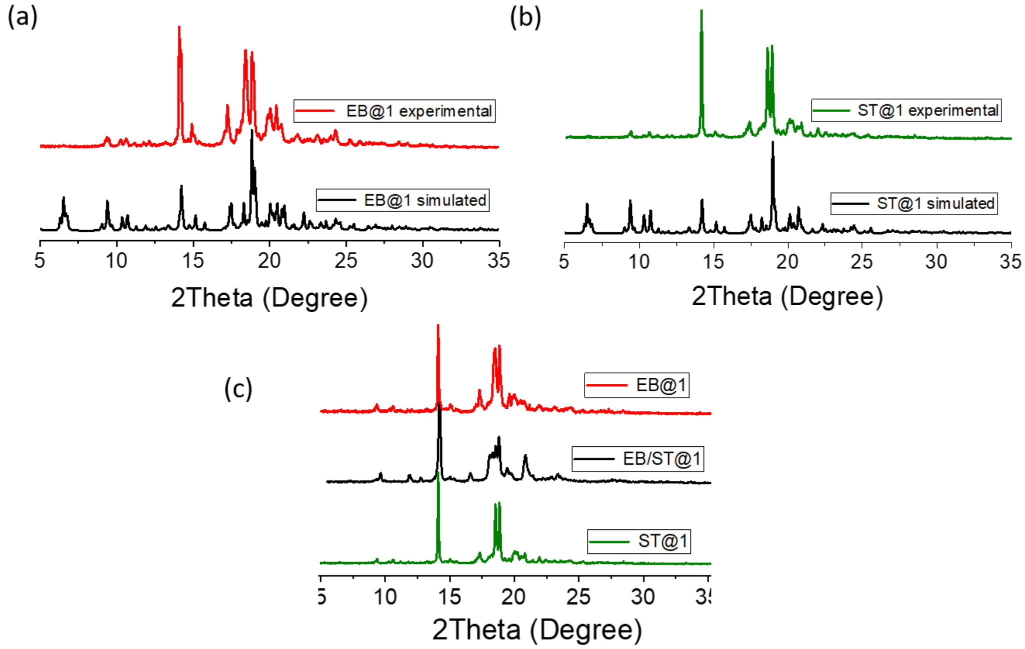

Figure S11. Comparison of simulated and experimental PXRD of (a) **EB@1**. (b) **ST@1**. (c) **EB@1**, **ST@1** and **ST/EB@1**.

a)

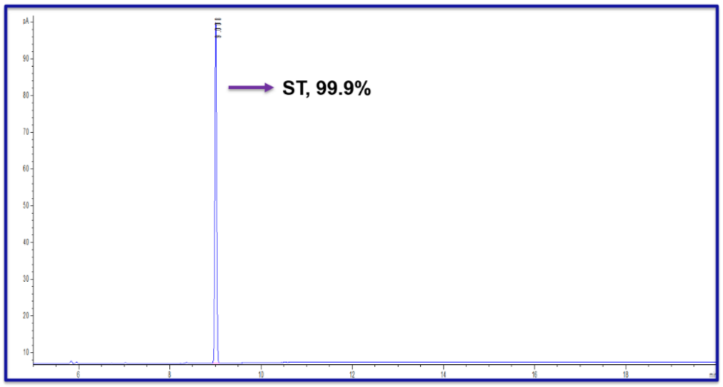

b)

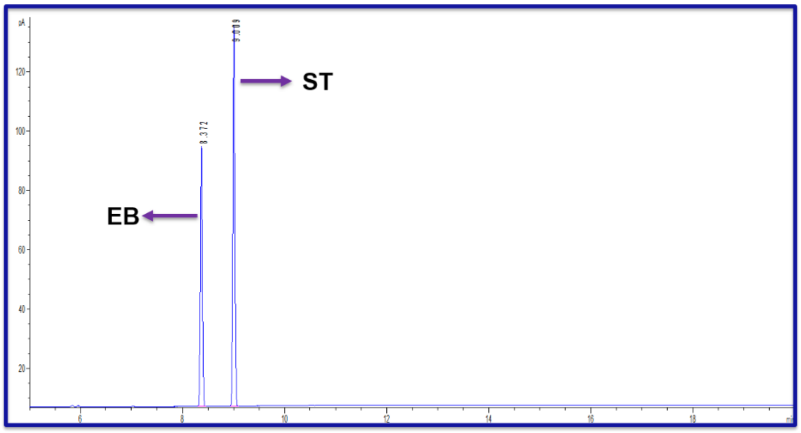

Figure S12. (a) GC analysis of the crystal obtained from ST/EB mixture which shows ST selectivity. (b) Reference GCMS where traces of known EB and ST present to quantify the ST peak.

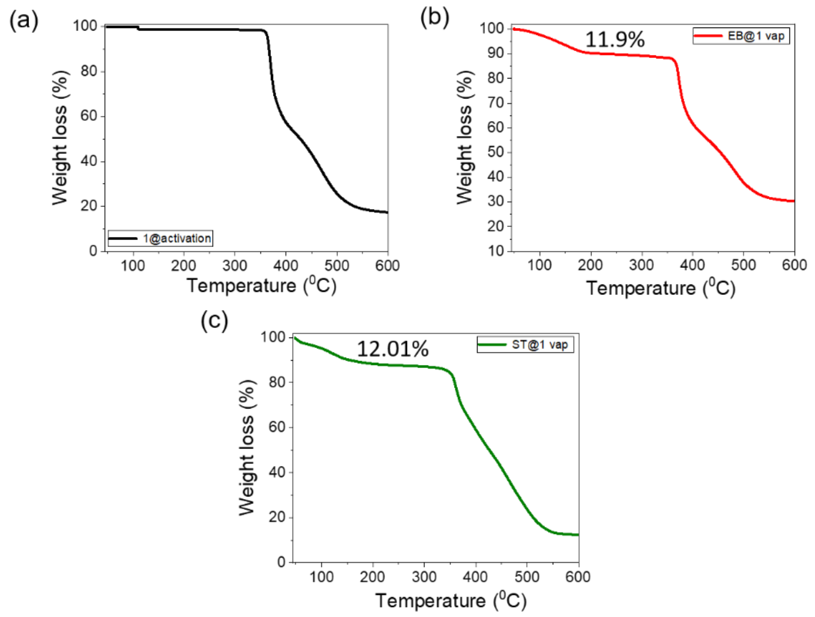

Figure S13. TGA of (a) Activated **1** at 70°C under high vacuum. (b) **EB@1** vapor. (c) **ST@1** vapor.

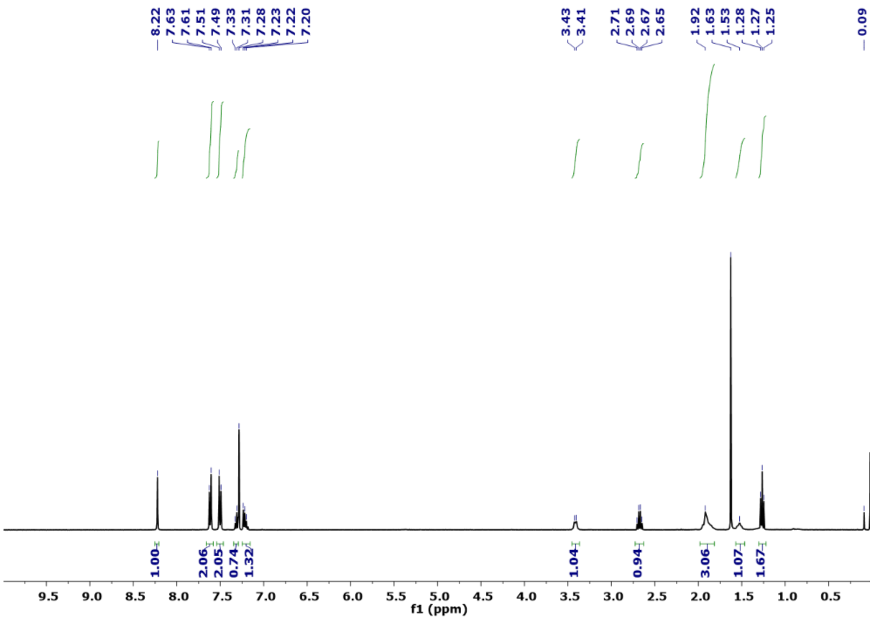

Figure S14. ^{1}H-NMR of **EB@1** in the solid-vapor experiment which shows uptake of almost 1 equiv. of EB.

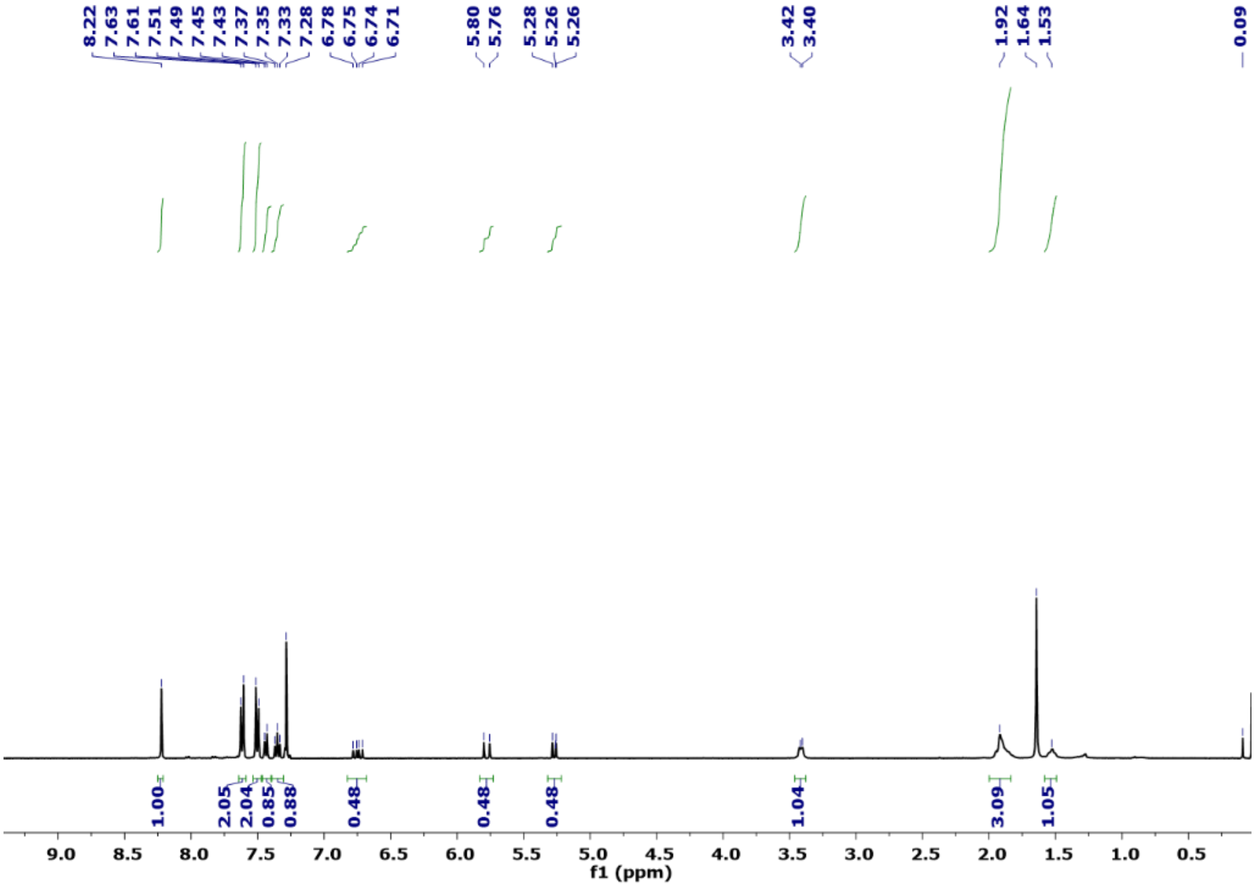

Figure S15. ^{1}H-NMR of **ST@1** in the solid-vapor experiment which shows which shows uptake of almost 1 equiv. of ST.

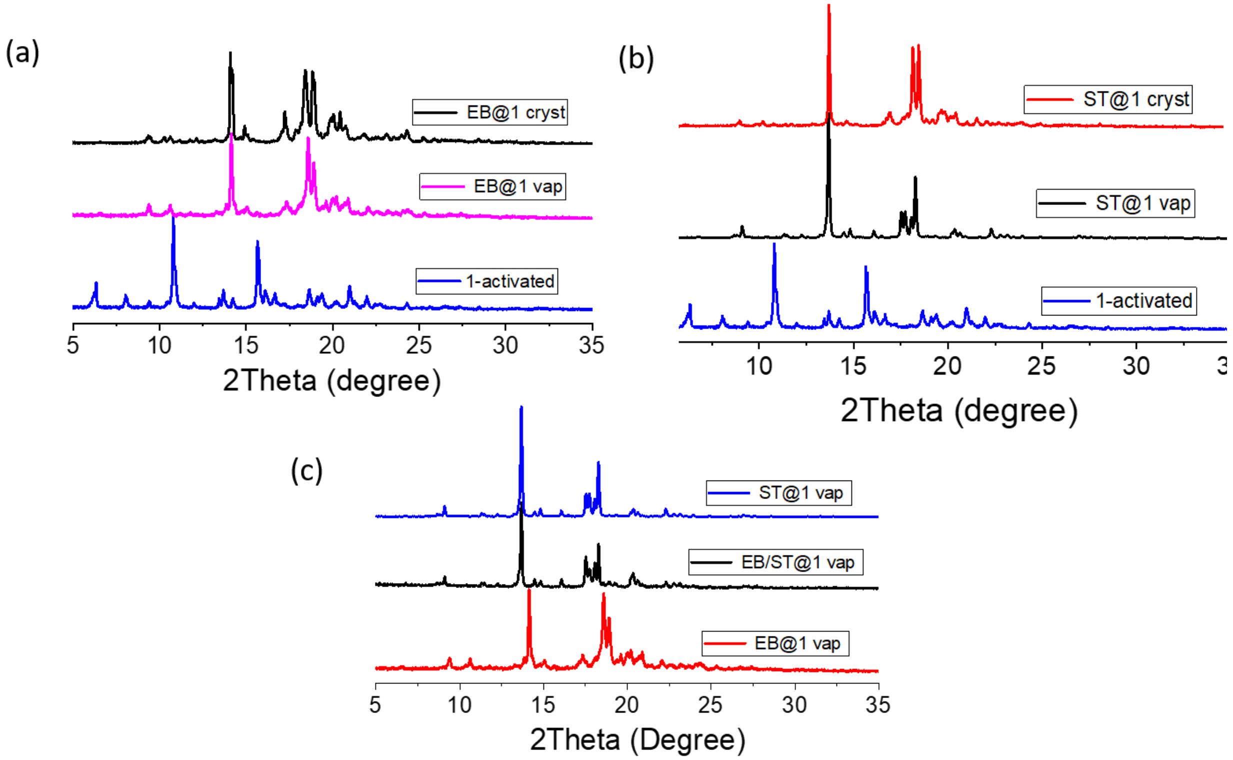

Figure S16. Comparison of PXRD. (a) **1**, **EB@1** obtained from EB vapor and crystal of **EB@1**. (b) **1**, **ST@1** obtained from ST vapor and crystal of **ST@1**. (c) **EB@1** obtained from EB vapor, **ST@1** obtained from ST vapor and **ST@1** obtained from EB/ST vapor.

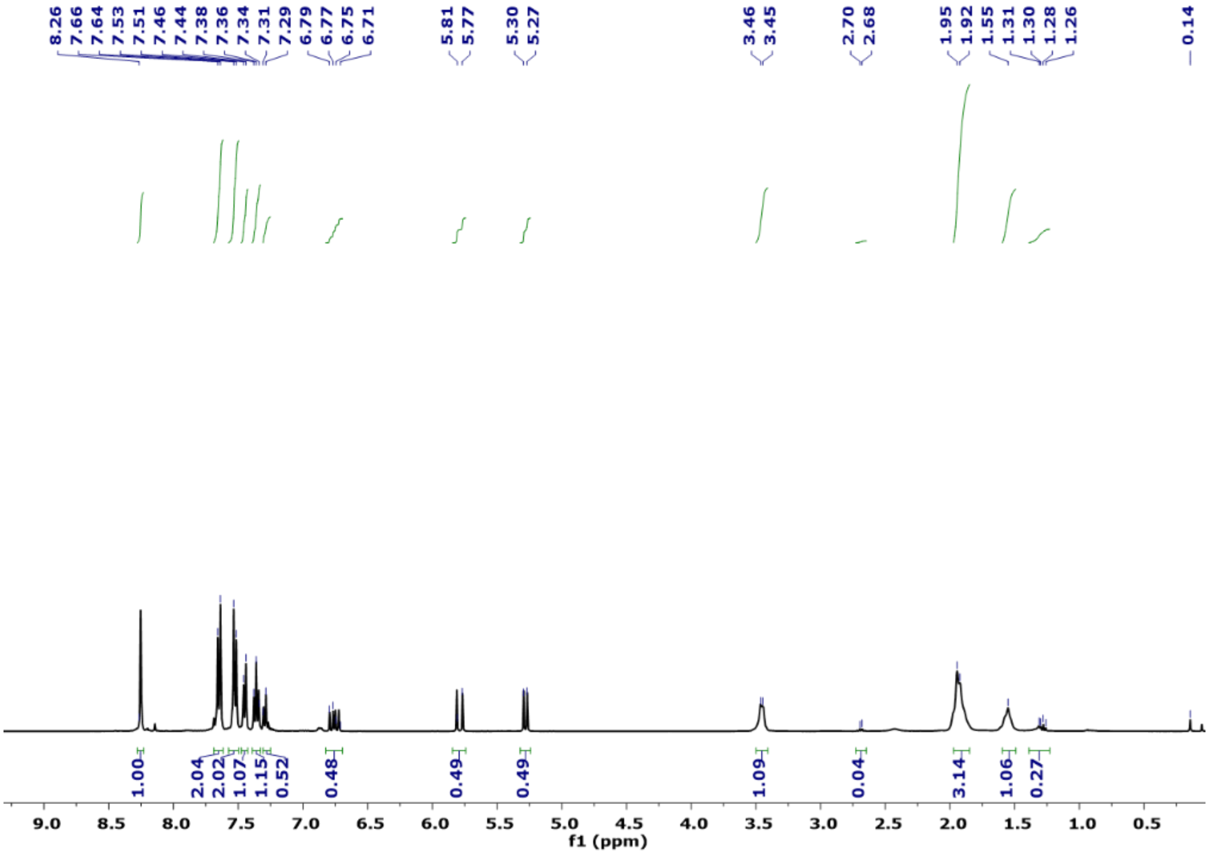

Figure S17. ^{1}H-NMR of **1** in the solid-vapor experiment of EB/ST mixture that shows ST selectivity.

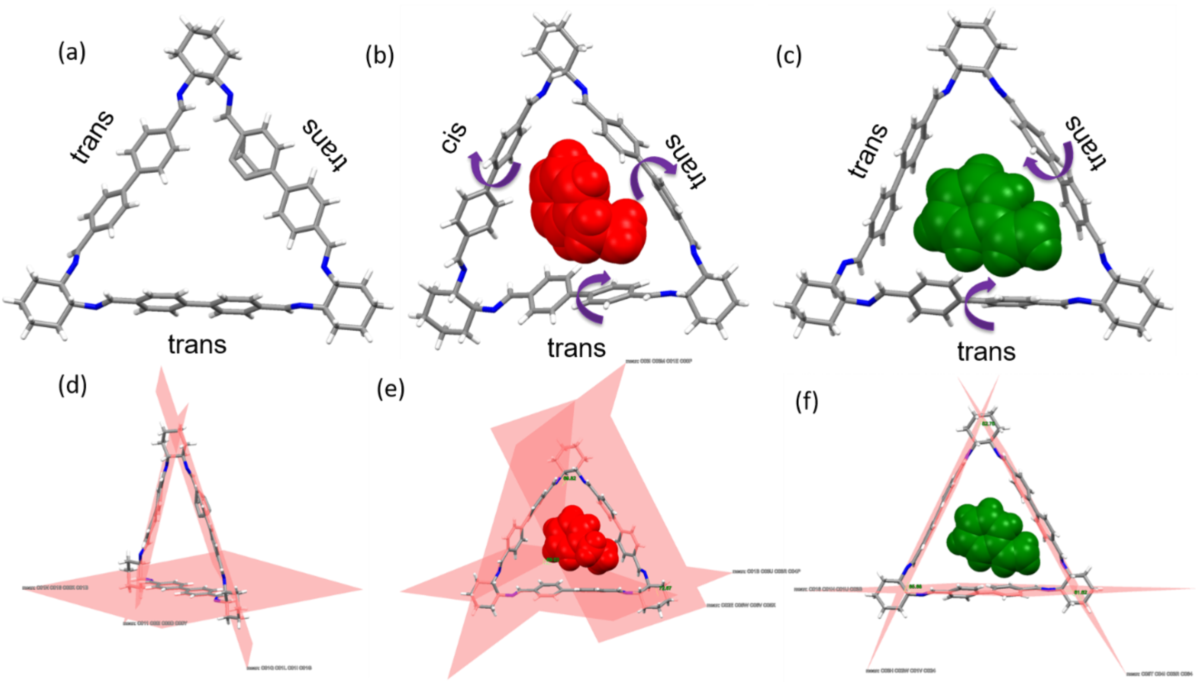

Figure S18. (a) All biphenyl moieties are in *trans* orientation with respect to (w.r.t) imine C=N bonds in **1**. (b) One biphenyl moiety is in *cis* orientation and other two biphenyl moieties are in *trans* orientation w.r.t C=N bonds in **EB@1** indicating change in orientation of the macrocyclic ring due to the presence of nonplanar ethyl group. (c) All the biphenyl moieties are in *trans* orientation w.r.t C=N bonds in **ST@1** indicating planarity giving macrocyclic ring stability. (d) Interplanar angle of the guest free structure **1**. (e) How the interplanar angle is changed after EB inclusion indicating nonplanar ethyl group making the adjustment of noncovalent interactions by C-C bond rotation. (f) Interplanar angle between the phenyl groups in **1** makes the easy accessibility for planar styrene which reflected in the selectivity.

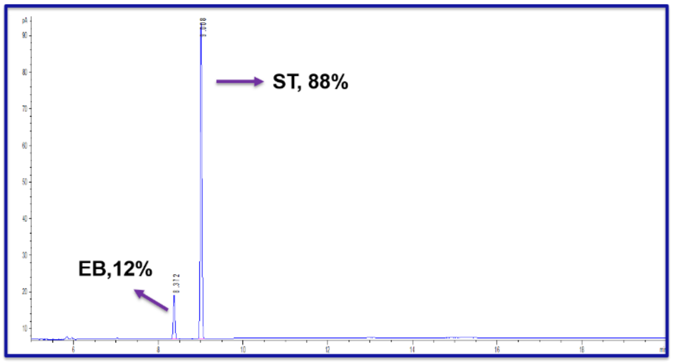

Figure S19. GC analysis of the crystalline materials obtained from ST/EB mixture vapor.

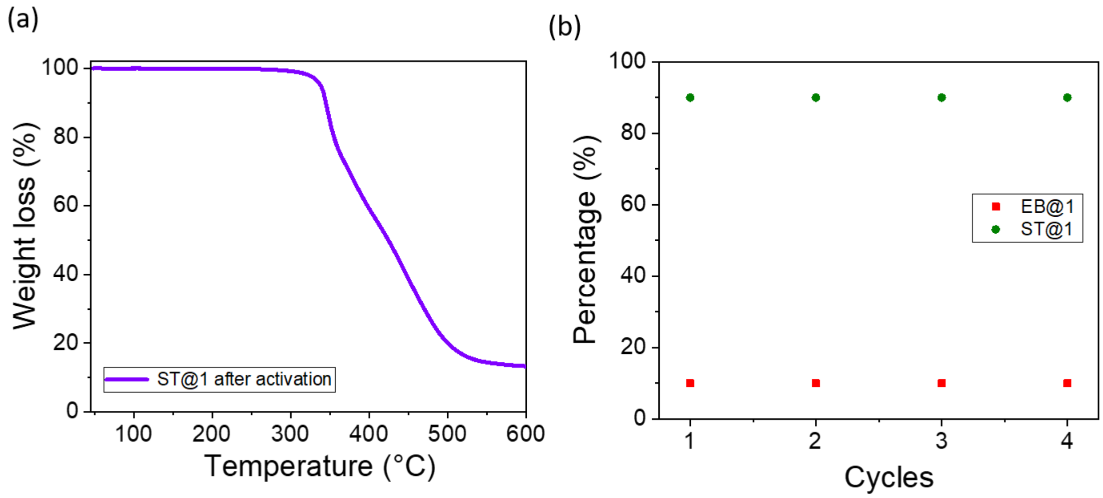

Figure S20. (a) Activation of **ST@1** at 100°C for 36 hrs under high vacuum shows that macrocycle was ST free. (b) Recyclability experiments in vapor phase shows the excellent stability and selectivity by GC after multiple cycles.

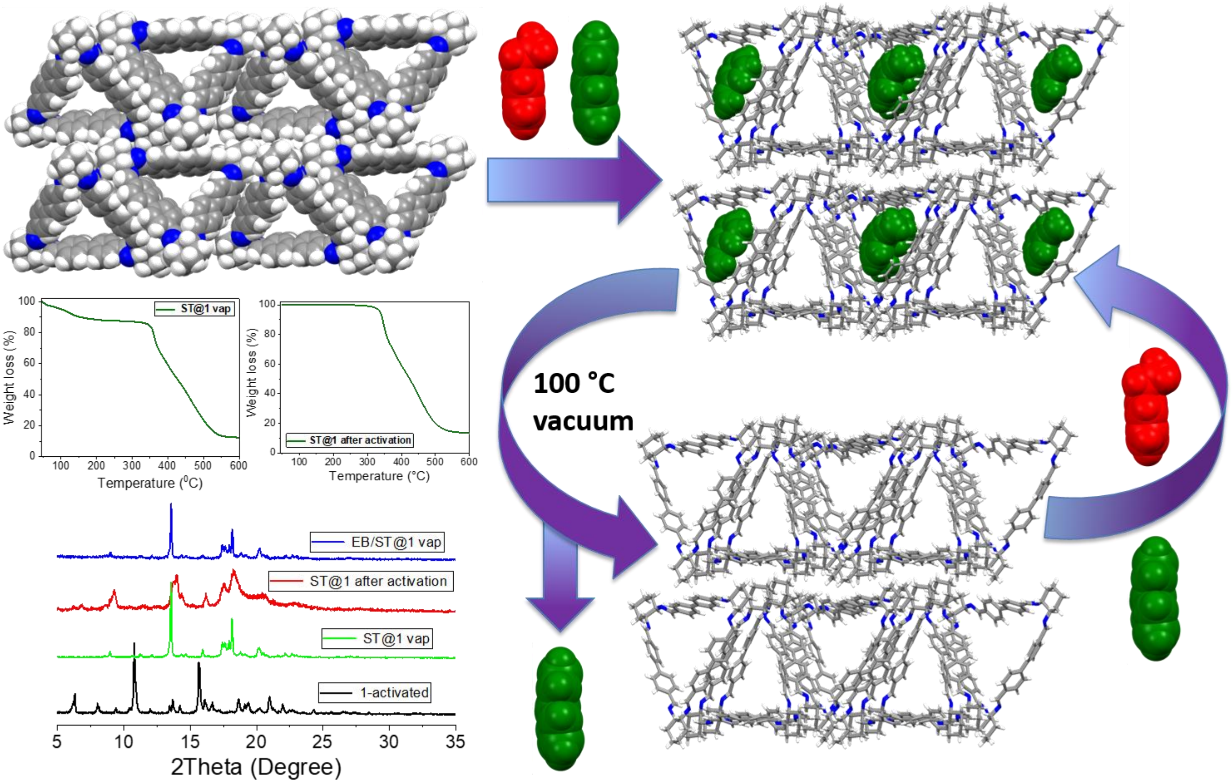

Figure S21. Recyclability experiments showing that the separation efficiency is well maintained.

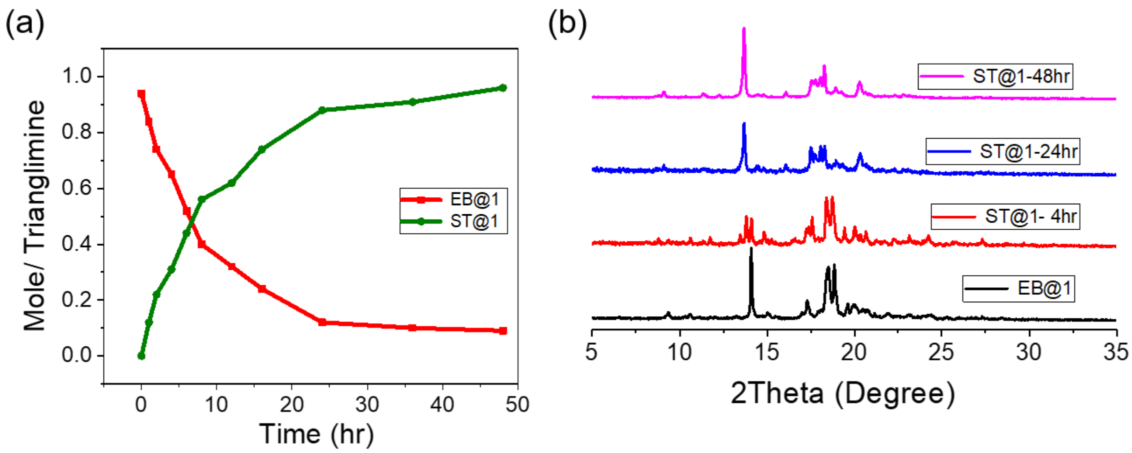

Figure S22. (a) Transformation of **EB@1** to **ST@1** over time when exposed to ST vapor. (b) Time dependent PXRD shows how **EB@1** transforms to **ST@1** over time when kept in ST vapor.

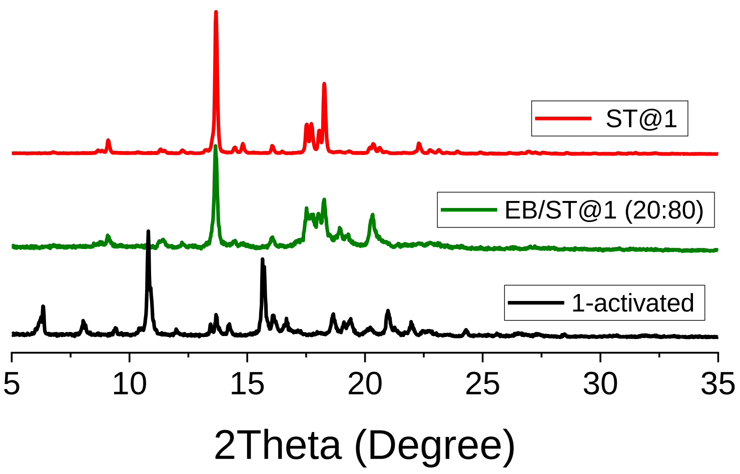

Figure S23. Comparison of PXRD of activated-**1**, **ST@1** and **ST@1** obtained from EB/ST (20:80%) mixture.

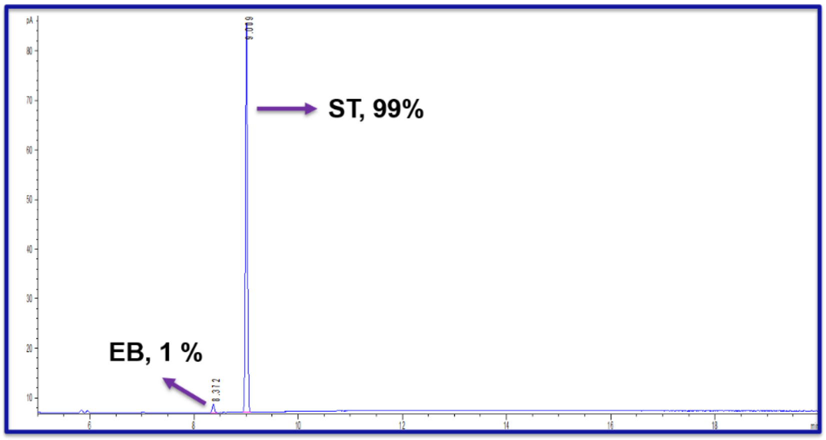

Figure S24. GC analysis of the obtained crystals of **ST@1** from EB/ST (20:80%) mixture in the solid-vapor experiment.

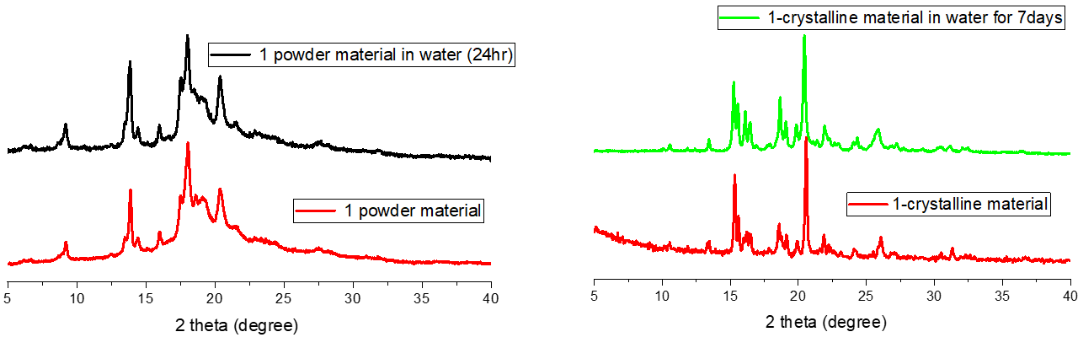

Figure S25. Stability of powder and crystalline macrocycle **1** in water.

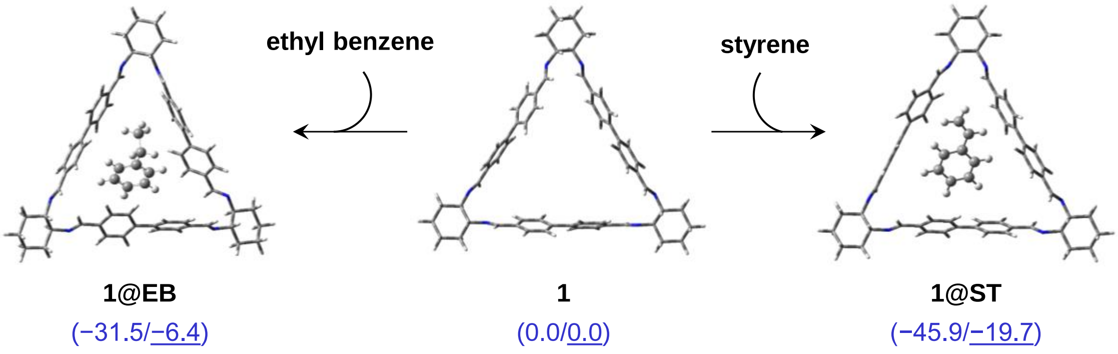

Figure S26. a) Energy values ($\Delta H_{298}^{Sol}/\Delta G_{298}^{Sol}$) are in kcal/mol at ωB97xD(SMD)/Def2-TZVP//PBE0-D3/Def2-SVP level of theory.

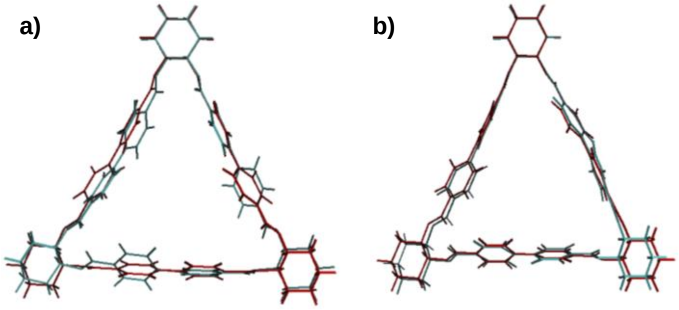

Figure S27. a) Superposition of DFT optimized geometry of **1** and its distorted geometry in **EB@1**. b) Superposition of DFT optimized geometry of **1** and its distorted geometry in **ST@1**.

Single Crystal X-ray Diffraction. Single crystals of the macrocycle trianglimine, were mounted in a Hampton cryoloop with *Paratone*® N oil cryoprotectant. In each case, a suitable crystal of appropriate size was selected from the mother liquor and immersed in *Paratone*® N oil and then it was mounted on the tip of a glass fiber and cemented using epoxy resin. Single crystal X-ray diffraction (SCXRD) was performed using a Bruker D8-Venture single crystal X-ray diffractometer equipped with a digital camera diffractometer using graphite-monochromated Mo-Kα radiation (0.71073 Å) at 120 K temperature. The linear absorption coefficients, scattering factors for the atoms and the anomalous dispersion corrections were taken from International Tables for X-ray Crystallography. Data integration and reduction were performed using SaintPlus 6.01[2] software. Absorption correction was performed by multi-scan method implemented in SADABS.[3] Space group was determined using XPREP implemented in APEX-III.[4] Structure was solved using Direct Methods (SHELXS-97)[5] and refined using SHELXL-2014[6] program package (full-matrix least squares on F^2) contained in WinGX.[7] For all the cases non-hydrogen atoms were refined anisotropically. All other hydrogen atoms are geometrically fixed using riding atom model. Attempts to identify the highly disordered solvent molecules have failed in some cases. Instead, a new set of F^2 (hkl) values with the contribution from the solvent molecules withdrawn was obtained by the SQUEEZE procedure implemented in PLATON.[8] The crystal data and refinement conditions for all the macrocycles trianglimine were collected in Table S1- S3.

Table S1. Crystal data and structure refinements for **EB@1**.

Identification code	**EB@1**
Empirical formula	$C_{128}H_{130}N_{12}$
Formula weight	1836.43
Temperature (K)	120(2)
Radiation	Mo-Kα
Wave length (λ)	0.71073
Crystal system	Triclinic
Space group	*P*1
a [Å]	15.730(2)
b [Å]	19.756(2)
c [Å]	20.229(3)
α [°]	81.276(4)

β [°]	68.667(4)
γ [°]	70.912(4)
Volume [$Å^3$]	5529.9(12)
Z	2
Density (calculated)[Mg m^{-3}]	1.103
Absorption coefficient [mm^{-1}]	0.065
F(000)	1964
Refl. used [$I > 2\sigma(I)$]	40903
Independent reflections	44443
R_{int}	0.0514
Refinement method	full-matrix least squares on F^2
GOF	1.038
Final *R* indices [I>2σ(I)]	R_1 = 0.0645; wR_2 = 0.0688
R indices (all data)	R_1 = 0.1715; wR_2 = 0.1753
CCDC number	2061331

Two EB solvent molecules are found to be disordered and could not be refined. The final refinement was performed using the PLATON SQUEEZE by removing the solvents. [6,8] The presence of solvent molecules could easily be seen by the residual peaks located in the open channels. The estimated electron count of two ethylbenzene (EB) solvent molecules (116 electrons; found 116.1) was considered per unit cell for **EB@1**. Taking into account the number of solvent molecules which were squeezed we determined host to guest ratio as 1:1.

Table S2. Crystal data and structure refinements for **ST@1**.

Identification code	**ST@1**
Empirical formula	$C_{248}H_{248}N_{24}$
Formula weight	3564.69
Temperature (K)	120(2)
Radiation	Mo-Kα
Wave length (λ)	0.71073

Crystal system	Triclinic
Space group	*P*1
a [Å]	15.6990(18)
b [Å]	19.838(2)
c [Å]	20.171(2)
α [°]	80.577(4)
β [°]	68.988(3)
γ [°]	70.374(4)
Volume [$Å^3$]	5516.9(11)
Z	1
Density (calculated)[Mg m^{-3}]	1.073
Absorption coefficient [mm^{-1}]	0.063
F(000)	1904
Refl. used [$I > 2\sigma(I)$]	27928
Independent reflections	42845
R_{int}	0.0831
Refinement method	full-matrix least squares on F^2
GOF	0.947
Final *R* indices [I>2σ(I)]	R_1 = 0.0705; wR_2 = 0.1032
R indices (all data)	R_1 = 0.1760; wR_2 = 0.1961
CCDC number	2061332

Three ST solvent molecules are found to be disordered and could not be refined. The final refinement was performed using the PLATON SQUEEZE by removing the solvents.[6,8] The estimated electron count of three styrene (ST) solvent molecules (168 electrons; found 178.0) was considered per unit cell for **ST@1**. Taking into account the number of solvent molecules which were squeezed we determined host to guest ratio as 1:1.

Table S3. Crystal data and structure refinements for **1**.

Identification code	**1**
Empirical formula	$C_{62}H_{58}N_6$
Formula weight	887.14
Temperature (K)	120(2)
Radiation	Mo-Kα
Wave length (λ)	0.71073
Crystal system	Monoclinic
Space group	$P2_1$
a [Å]	17.045(2)
b [Å]	5.436(5)
c [Å]	28.076(2)
α [°]	90
β [°]	91.47(5)
γ [°]	90
Volume [Å^3]	2600.8(3)
Z	2
Density (calculated)[Mg m^{-3}]	1.133
Absorption coefficient [mm^{-1}]	0.067
F(000)	944
Refl. used [$I > 2\sigma(I)$]	9085
Independent reflections	11796
R_{int}	0.0724
Refinement method	full-matrix least squares on F^2
GOF	1.045
Final *R* indices [I>2σ(I)]	R_1 = 0.0828; wR_2 = 0.2268
R indices (all data)	R_1 = 0.1057; wR_2 = 0.2440
CCDC number	2061333

Computational Details

All the geometries were optimized with Gaussian 16 program packages,[9] using hybrid generalized gradient approximation (h-GGA) DFT functional PBE0.[10] To account for dispersion effects, empirical dispersion-corrected model at GD3 level were applied.[11] The electronic configuration of all atoms were described with the Ahlrichs split-valance polarization basis function Def2-SVP[12] The geometries were optimized without any symmetry constraints. Harmonic force constants were computed at the optimized geometries to characterize the stationary points as minima or saddle points. For further validation of energetics, single-point calculations were performed on the PBE0-D3/Def2-SVP optimized geometries using the long-range corrected hybrid density functional, ωB97xD, with damped atom−atom dispersion corrections[13] employing a valence triple-ζ-type of basis set Def2-TZVP[14]. The solvent effects (ethyl benzene, ε = 2.4339) were evaluated implicitly by a self-consistent reaction field (SCRF) approach using the SMD continuum solvation model.[15] The rigid-rotor harmonic-oscillator approximation was applied for evaluating the thermal and entropic contributions that are needed to derive the enthalpies and Gibbs free energies. The ΔH_{298}^{Sol} value was obtained by augmenting the ΔE_{e}^{Sol} energy terms at ωB97xD(SMD)/Def2-TZVP with the respective enthalpy corrections at the PBE0-D3/Def2-SVP level in the gas phase. Since the thermal correction to the Gibbs energy of each component depends on its concentration in solution, one can incorporate the concentration terms into calculations. The Gibbs free energy corrections of each component is modified by incorporating its concentration in solution in terms of partial pressure. In the Gaussian program, the concentration can be specified by adjusting the pressure value based on the ideal gas law $p_i = (n_i/V)RT$, where p_i is the partial pressure, R the gas constant (0.082 L·atm·K^{-1}·mol^{-1}), T the absolute temperature, n_i the molar quantity, and V the reaction volume. The experimental concentrations of host and guest are approximated by setting the partial pressures as follows:

component	n_i (mol)	V (L)	R (L·atm·K^{-1}·mol^{-1})	T (K)	p_i (atm)
**1**	1.1x10^{-5}	1.0x10^{-3}	0.082	298.15	0.27
ST,<br>EB	7.2x10^{-3}				1.76x10^{2}

The Gibbs free energy in the solution phase is a rough approximation, which is not appropriate for association and dissociation processes because there is a significant degree of denial of the translational degrees of freedom upon moving from the gas to solution phase. As a result, the Sackur–Tetrode equation, which is generally used to

calculate the gas-phase translational entropy, cannot be applied directly in the solution phase. Therefore, the solvation entropy (S_{298}^{Sol}) has been estimated as two-thirds of the gas-phase value.[16] Finally, the ΔG_{298}^{Sol} was calculated as: $\Delta G_{298}^{Sol} = \Delta H_{298}^{Sol} - T\Delta S_{298}^{Sol}$. Here, ΔS_{298}^{Sol} represents the solvation entropy, which was estimated as 2/3 of the gas phase value.

Activation strain-distortion/interaction analysis

In this method the bond dissociation energy D_e of a complex AB is divided into the instantaneous interaction energy (ΔE_{int}) and the distortion energy (ΔE_{dis}) according to eq 1.

$$\Delta E\ (= -D_e) = \Delta E_{int} + \Delta E_{dis} \qquad (1)$$

The ΔE_{dis} is the energy that required to promote fragments A and B from their equilibrium geometries in the electronic ground state to the geometries in the corresponding intermediates/complexes, whereas ΔE_{int} is the actual interaction energy between the prepared fragments in the respective intermediates/complexes. The ΔE_{int} can be further divided into different components as explain in the following ETS-NOCV analysis.

Energy Decomposition Analysis (EDA)

We have performed the energy decomposition analysis (EDA) using the program package ADF 2019.301.[17] All analyses were performed by employing the BLYP[18] functional with triple-ξ-quality basis set using uncontracted Slater-type orbitals (STOs) augmented by two sets of polarization functions (TZ2P) for all atoms, with no frozen-core approximation for inner core electrons.[19] To compute the dispersion effects, we have utilized Grimme's D3BJ[20] empirical correction. The EDA method, developed independently by Morokuma[21] and Ziegler and Rauk,[22] gives a quantitative description of the chemical bonds in molecules.

In this method the ΔE_{int} can be divided into three main components:

$$\Delta E_{int} = \Delta E_{elstat} + \Delta E_{Pauli} + \Delta E_{orb} \qquad (2)$$

In eq 2, ΔE_{elstat} is the quasiclassical electrostatic interaction energy between the fragments. ΔE_{Pauli} refers to the repulsive interactions between the fragments, which are caused by the fact that two electrons with the same spin cannot occupy the same region in space and can be calculated by enforcing the Kohn−Sham determinant on the superimposed fragments to obey the Pauli principle by antisymmetrization and renormalization. The stabilizing orbital interaction term ΔE_{orb} is calculated in the final step of the energy partitioning analysis when the Kohn−Sham orbitals relax to their optimal form.

Table S4. Distortion/interaction analysis on **EB@1** and **ST@1**. The energy terms (in kJ/mol) ΔE_{dis} (distortion energy), ΔE_{int} (interaction energy) and ΔE (ΔE_{dis} + ΔE_{int}) are calculated at the ωB97xD(SMD)/Def2-TZVP level of theory.

	**EB@1**	**ST@1**
ΔE_{dis}	18.7(1), 0.4(EB)	2.1(1), 0.3(ST)
ΔE_{int}	−56.0	−54.0
ΔE_{bond}	−36.8	−51.7

Table S5. Results of EDA calculations for **EB@1** and **ST@1** at BLYP-D3/TZ2P level.

	**EB@1**	**ST@1**
ΔE_{int}	−75.3	−74.3
ΔE_{elstat}	−39.2	−42.2
ΔE_{Pauli}	99.6	103.5
ΔE_{orb}	−20.7	−23.7
ΔE_{disp}	−115.0	−111.9

Explanation of crystallographic alerts of **ST@1**:

1. *PLAT331_ALERT_2_A Small Aver Phenyl C-C Dist C078 --C07K. 1.34 Ang.*

Response: This is because of C078 atom is not ideally shaped, however, this does not indicate an incorrect atom-type assignment. We observed deviations of expected thermal parameters which are in agreement with a slight rotational disorder of this particular carbon atom.

2. *PLAT410_ALERT_2_A Short Intra H...H Contact H99...H07K. 1.73 Ang. x,y,z = 1_555 Check*

Response: This is due to the guest ST molecule is not ideally shaped, however, this does not indicate an incorrect atom-type assignment. We observed deviations of expected thermal parameters which are in agreement with a slight rotational disorder of this ST molecule.

References:

1) Jie, K.; Liu, M.; Zhou, Y.; Little, M. A.; Bonakala, S.; Chong, S. Y.; Stephenson, A.; Chen, L.; Huang, F.; Cooper, A. I. *J. Am. Chem. Soc*. **2017**, *139*, 2908−2911.

2) SAINT. Bruker AXS. Inc, Madison, Wisconsin, USA, **2014**.

3) SADABS. G. M. Sheldrick. University of Gottingen, Germany, **2008**.

4) XPREP, 5.1 ed. Siemens Industrial Automation Inc., Madison, WI, **1995**.

5) Sheldrick, G. M. *SHELXTL* ™*Reference Manual*: version 5.1, Bruker AXS, Madison, WI, **1997**.

6) Sheldrick, G. M. Crystal Structure Refinement with SHELXL. *Acta Cryst C*, **2015**, *71*, 3–8.

7) WinGX. L. J. Farrugia, *J. Appl. Cryst*. **2012**, *45*, 849-854.

8) Spek, A. L. Single-crystal Structure Validation with the Program PLATON. *J. Appl. Crystallogr*. **2003**, *36*, 7−13.

9) Gaussian 16, Revision **B.01**, M. J. Frisch, G. W. Trucks, H. B. Schlegel, G. E. Scuseria, M. A. Robb, J. R. Cheeseman, G. Scalmani, V. Barone, G. A. Petersson, H. Nakatsuji, X. Li, M. Caricato, A. V. Marenich, J. Bloino, B. G. Janesko, R. Gomperts, B. Mennucci, H. P. Hratchian, J. V. Ortiz, A. F. Izmaylov, J. L. Sonnenberg, D. Williams-Young, F. Ding, F. Lipparini, F. Egidi, J. Goings, B. Peng, A. Petrone, T. Henderson, D. Ranasinghe, V. G. Zakrzewski, J. Gao, N. Rega, G. Zheng, W. Liang, M. Hada, M. Ehara, K. Toyota, R. Fukuda, J. Hasegawa, M. Ishida, T. Nakajima, Y. Honda, O. Kitao, H. Nakai, T. Vreven, K. Throssell, J. A. Montgomery, Jr., J. E. Peralta, F. Ogliaro, M. J. Bearpark, J. J. Heyd, E. N. Brothers, K. N. Kudin, V. N. Staroverov, T. A. Keith, R. Kobayashi, J. Normand, K. Raghavachari, A. P. Rendell, J. C. Burant, S. S. Iyengar, J. Tomasi, M. Cossi, J. M. Millam, M. Klene, C. Adamo, R. Cammi, J. W. Ochterski, R. L. Martin, K. Morokuma, O. Farkas, J. B. Foresman, and D. J. Fox, Gaussian, Inc., Wallingford CT, **2016**.

10) Adamo, C.; Barone, V. *J. Chem. Phys*. **1999**, *110*, 6158−6169.

11) Grimme, S.; Antony, J.; Ehrlich S.; Krieg, H. *J. Chem. Phys*. **2010**, *132*, 154104.

12) Schäfer, A.; Horn, H.; Ahlrichs, R. *J. Chem. Phys*. **1992**, *97*, 2571−2577.

13) Chai, J.-D.; Head-Gordon, M. *Phys. Chem. Chem. Phys*. **2008**, *10*, 6615−6620.

14) Schaefer, A.; Huber, C.; Ahlrichs, R. *J. Chem. Phys*. **1994**, *100*, 5829−5835.

15) Marenich, A. V.; Cramer, C. J.; Truhlar, D. G. *J. Phys. Chem*. *B* **2009**, *113*, 6378−6396.

16) (a) Cooperand J.; Ziegler, T. *Inorg. Chem*. **2002**, *41*, 6614–6622; (b) Tobisch, S. *Chem.–Eur. J*. **2005**, *11*, 3113–3126; (c) Tommaso, S. D.; Tognetti, V.; Sicilla, E.; Adamo, C.; Russo, N. *Inorg. Che*m. **2010**, *49*, 9875–9883.

17) G. te Velde, F. M. Bickelhaup, E. J. Baerends, C. Fonseca Guerra, S. J. A. Van Gisbergen, J. G. Snijders, T. Ziegler, *J. Comput. Chem*. **2001**, *22*, 931−967.

18) a) A. D. Becke, *Phys. Rev. A* **1988**, *38*, 3098–3100; b) C. Lee, W. Yang, R. G. Parr, *Phys. Rev. B* **1988**, *37*, 785–789; c) B. Miehlich, A. Savin, H. Stoll, H. Preuss, *Chem. Phys. Lett*. **1989**, *157*, 200–206.

19) a) E. Clementi, C. Roetti, *Data Nucl. Data Tables* **1974**, *14*, 177−478; b) A. D. McLean, R. S. McLean, *Data Nucl. Data Tables* **1981**, *26*, 197−381; c) J. G. Snijders, P. Vernooijs, E. J. Baerends, *Data Nucl. Data Tables* **1981**, *26*, 483−509; d) D. P. Chong, E. V. Lenthe, S. V. Gisbergen, E. J. Baerends, *J. Comput. Chem*. **2004**, *25*, 1030−1036.

20) S. Grimme, S. Ehrlich, L. Goerigk, *J. Comp. Chem*. **2011**, *32*, 1456−65.

21) a) K. Morokuma, *J. Chem. Phys*. **1971**, *55*, 1236−1244; b) K. Kitaura, K. Morokuma, *Int. J. Quantum Chem*. **1976**, *10*, 325−340.

22) a) T. Ziegler, A. Rauk, *Inorg. Chem*. **1979**, *18*, 1755−1759; b) T. Ziegler, A. Rauk, *Theor. Chim. Acta* **1977**, *46*, 1−10.

Cartesian coordinates (Å) of the optimized structures of all intermediates and transition states at PBE0-D3/Def2-SVP level of theory. EeS represents the absolute electronic energy in Hartree at M06(SMD)/Def2-TZVP level of theory in EB and ST solvent.

1

126

E_e^{Sol}: -2651.15604989

N	12.794862	16.413759	16.311445
N	4.805211	4.884418	19.848494
N	2.222558	15.361760	24.275147
N	13.235562	13.920121	15.003802
N	3.795378	17.614619	23.586627
N	6.951000	4.606036	18.022324
C	13.749864	15.153124	14.477068
H	13.008312	15.665076	13.825654
C	9.451191	10.991331	15.636111
H	8.391280	10.803134	15.449884
C	2.483065	17.767142	24.152112
H	1.699538	17.762638	23.362733
C	10.049342	12.133458	15.114166
H	9.458615	12.827641	14.509352
C	8.034206	6.995699	16.780234
H	7.315301	6.407782	16.205336
C	12.577511	15.946066	17.470945

H	13.336062	15.336276	18.003848
C	3.984883	5.795860	19.523948
H	3.345511	5.706317	18.620519
C	10.183071	10.096761	16.430629
C	14.036228	16.111190	15.655873
H	14.741369	15.580015	16.331863
C	4.917405	3.721599	19.010810
H	4.401766	3.869133	18.036981
C	12.015784	13.626641	14.812992
H	11.344838	14.283862	14.222140
C	9.553637	8.884109	16.994636
C	8.882434	16.542263	19.575055
C	11.397386	12.413933	15.364726
C	9.935983	15.820773	20.155300
H	9.804571	15.378278	21.145469
C	6.406617	3.467451	18.709746
H	6.900540	3.316044	19.694733
C	9.294732	7.296224	18.813269
H	9.553928	6.971555	19.825288
C	11.131196	15.630562	19.469801
H	11.936091	15.051782	19.932201
C	5.167149	17.146083	21.660111
C	9.071943	17.069755	18.284183
H	8.273930	17.657511	17.824049
C	7.606628	16.743241	20.293890
C	2.387022	19.072476	24.939519
H	3.186013	19.076588	25.701140
H	2.599063	19.914413	24.261311
C	8.615170	8.140399	16.255504
H	8.362882	8.455858	15.240132
C	2.181188	16.562700	25.063976
H	2.951842	16.551256	25.865250
C	9.880429	8.440614	18.284607
H	10.584000	9.016627	18.890047
C	3.887486	17.360581	22.346686
H	2.985106	17.284163	21.705220
C	8.368896	6.555762	18.069207
C	6.383703	16.708187	19.607779
H	6.373374	16.498488	18.535734
C	11.312963	16.159746	18.186995
C	15.025617	14.888431	13.677871
H	14.782996	14.222821	12.834045
H	15.723716	14.329559	14.324832
C	10.264258	16.884783	17.602343
H	10.419904	17.300620	16.604468
C	3.501553	9.442280	21.748209
C	7.772937	5.344699	18.646258
H	8.088119	5.107931	19.683662
C	11.537523	10.386433	16.679726
H	12.133019	9.689319	17.274217
C	3.341642	10.704775	22.501993
C	1.022379	19.230848	25.598464
H	0.247752	19.326491	24.816386
H	0.989425	20.164303	26.182932
C	2.581046	13.015839	22.617970
H	2.113132	13.890060	22.160296
C	15.673231	16.178086	13.190766
H	16.600284	15.950437	12.640473
H	14.999854	16.678946	12.471991
C	5.183791	16.903930	20.281649
H	4.239102	16.862342	19.731557
C	7.582242	16.978027	21.681137
H	8.524940	17.031388	22.231248
C	15.956341	17.122162	14.353913
H	16.386918	18.068897	13.990087
H	16.717216	16.667703	15.013819

C	3.821192	7.033933	20.296442
C	2.601418	9.084686	20.732272
H	1.747099	9.728720	20.513870
C	4.719369	7.377368	21.317031
H	5.548002	6.697350	21.525886
C	0.803436	16.724902	25.710365
H	0.615937	15.860855	26.368047
H	0.040322	16.683467	24.913849
C	14.690698	17.398456	15.154964
H	14.902126	18.047741	16.019645
H	13.953875	17.936676	14.533954
C	6.575006	2.202463	17.870487
H	7.647899	2.042269	17.678084
H	6.104013	2.374392	16.887081
C	2.918705	14.383693	24.684734
H	3.480108	14.431496	25.641549
C	2.733776	11.831052	21.915127
H	2.400453	11.782809	20.876202
C	4.307272	2.503955	19.709069
H	3.240516	2.702446	19.901238
H	4.788145	2.393938	20.696570
C	12.135561	11.521746	16.156176
H	13.188916	11.745834	16.338164
C	2.765683	7.907437	20.011551
H	2.053758	7.652692	19.220961
C	5.954562	0.989968	18.553562
H	6.063383	0.098182	17.915674
H	6.506218	0.773977	19.486232
C	4.566014	8.561807	22.020337
H	5.303846	8.827806	22.780372
C	3.797061	10.823011	23.824419
H	4.246615	9.961113	24.321776
C	3.049039	13.127755	23.935070
C	6.385947	17.178427	22.353324
H	6.362892	17.374083	23.427646
C	4.486207	1.234386	18.885170
H	4.061833	0.372378	19.424642
H	3.912593	1.326348	17.945386
C	0.694938	18.033333	26.484158
H	-0.314489	18.133784	26.914446
H	1.396133	18.010984	27.337684
C	3.663598	12.016908	24.523823
H	4.030680	12.086104	25.552036

Ethylbenzene (EB)

18

E_e^{Sol}: -310.890748868 A.U.

C	2.899859	10.095234	20.089693
H	2.087156	10.811428	20.233812
C	4.995176	8.249827	19.727299
C	4.205917	10.544008	19.896874
H	4.421173	11.615685	19.890021
C	5.241173	9.628170	19.717119
H	6.264001	9.988298	19.569875
C	2.639692	8.725374	20.102163
H	1.620037	8.363161	20.257012
C	3.678842	7.814064	19.921923
H	3.466992	6.740542	19.936574
C	6.288006	6.943354	17.997642
H	7.104548	6.221548	17.840852
H	5.367492	6.514374	17.572174
H	6.522296	7.853047	17.423220
C	6.107183	7.264244	19.480055
H	7.049559	7.666321	19.887164

H	5.901897	6.334320	20.035528

Styrene (ST)

16

E_e^{Sol}: -309.653583868

C	6.813114	11.006451	19.103976
C	6.649543	9.689293	18.473675
H	7.487344	9.381446	17.836840
C	5.611903	8.852504	18.594728
H	4.737478	9.083342	19.209775
H	5.603127	7.892446	18.073464
C	7.981944	11.741324	18.851828
H	8.744254	11.317129	18.192051
C	5.849071	11.573553	19.955181
H	4.927803	11.028135	20.172671
C	7.219259	13.541889	20.266259
H	7.373959	14.524679	20.718066
C	6.049963	12.824002	20.527973
H	5.286808	13.245734	21.186930
C	8.185590	12.994623	19.425015
H	9.104840	13.546451	19.212568

1@EB

144

E_e^{Sol}: -2962.06082980

N	-2.141408	6.211643	24.479868
N	8.618111	5.273873	14.414962
N	3.288345	15.636250	20.906631
N	-1.688252	3.861123	22.961682
N	7.246833	3.129181	15.672054
C	-0.215507	9.727772	23.012311
H	-0.727721	10.424481	22.344895
N	5.667576	15.522534	19.087951
C	6.012470	3.540478	17.689301
C	3.985878	16.765417	20.342611
H	3.484951	16.968610	19.376572
C	2.102693	3.406180	21.046405
H	2.834404	2.668096	21.384095
C	1.121378	9.975973	23.377143
C	-0.884222	8.597796	23.455546
H	-1.919560	8.400464	23.168826
C	6.025063	3.536337	19.088698
H	6.966443	3.364758	19.618759
C	1.791946	13.497212	22.156177
H	1.254268	14.424096	21.944832
C	0.896104	3.530349	21.718060
H	0.664677	2.917037	22.591678
C	-2.950298	4.109306	23.602340
H	-3.624836	4.714610	22.958797
C	4.801763	3.789811	17.027077
H	4.801237	3.784899	15.935073
C	1.761276	9.037404	24.200297
H	2.791398	9.211950	24.517243
C	-0.922404	6.452480	24.739427
H	-0.299408	5.737580	25.315921
C	7.548254	5.956818	14.405669
H	6.619806	5.578388	13.931824
C	-3.636567	2.791820	23.955611
H	-2.932346	2.190407	24.556162
H	-3.816202	2.225301	23.027871

C	7.850152	11.309669	18.196450
H	8.551970	10.552030	18.551680
C	8.538761	1.500561	14.413679
H	7.632775	1.344074	13.802922
H	8.481185	0.782220	15.247264
C	1.095226	7.899270	24.639334
H	1.613309	7.180660	25.281075
C	-0.236563	7.665086	24.279078
C	-2.701327	4.951753	24.877754
H	-1.998957	4.369235	25.513383
C	3.875028	12.285336	22.200031
H	4.949558	12.237908	22.000288
C	-4.010043	5.165140	25.637294
H	-4.670003	5.790352	25.011379
H	-3.800125	5.748762	26.547951
C	4.855460	3.750175	19.811022
H	4.886774	3.752606	20.903155
C	1.136076	12.375279	22.638326
H	0.063342	12.428530	22.837807
C	7.245859	3.294296	16.929758
H	8.183730	3.264783	17.522959
C	-0.074862	4.438838	21.272202
C	1.416052	5.103767	19.477506
H	1.626994	5.748128	18.621259
C	6.542349	14.641353	19.346973
H	7.173917	14.702919	20.260145
C	-1.370363	4.562922	21.952567
H	-2.060986	5.321944	21.531590
C	8.598088	3.955844	13.842782
H	7.701767	3.801625	13.203668
C	3.213275	11.155292	22.663276
H	3.769731	10.224881	22.792665
C	-4.933507	3.012551	24.723365
H	-5.660520	3.532953	24.073974
H	-5.388789	2.043459	24.983449
C	1.830709	11.178715	22.896210
C	8.569368	7.884447	15.624566
H	9.524588	7.357486	15.574265
C	9.790038	1.267170	13.576317
H	10.681882	1.332564	14.225242
H	9.780767	0.245514	13.163698
C	3.639929	3.992031	19.153924
C	2.380821	4.179340	19.904273
C	3.754487	17.980978	21.246884
H	4.238561	17.784296	22.220807
H	2.675226	18.062796	21.447155
C	5.483953	16.610236	20.014872
H	6.043743	16.437069	20.962253
C	-4.694174	3.847135	25.976894
H	-4.060587	3.275095	26.678561
H	-5.643266	4.039275	26.502643
C	3.640271	4.019743	17.747206
H	2.700853	4.189486	17.215999
C	0.207610	5.231531	20.153521
H	-0.531264	5.961291	19.809755
C	7.448816	7.278646	15.037608
C	8.489392	2.920324	14.983366
H	9.363718	3.071041	15.653408
C	3.891461	14.628832	21.386411
H	4.990742	14.531406	21.408741

C	3.175382	13.467744	21.933296
C	7.686250	12.483458	18.922355
H	8.275371	12.646843	19.829621
C	9.856360	3.715398	13.012789
H	9.895338	4.460287	12.201979
H	10.732955	3.908595	13.655229
C	6.753891	13.447489	18.522454
C	4.307356	19.262893	20.639193
H	4.155044	20.107078	21.330749
H	3.740302	19.503851	19.722298
C	7.086454	11.062852	17.045135
C	6.104435	9.179307	15.717285
H	5.124372	9.655254	15.792716
C	6.015484	17.906236	19.391963
H	7.087189	17.777256	19.170682
H	5.511021	18.050264	18.420575
C	8.455621	9.116732	16.247051
H	9.343653	9.582962	16.679937
C	9.907159	2.297573	12.458441
H	10.837764	2.146239	11.888337
H	9.077587	2.153294	11.742915
C	7.219356	9.785614	16.315788
C	5.783798	19.112697	20.291831
H	6.163737	20.024285	19.803104
H	6.367135	18.992879	21.222658
C	6.219953	7.946104	15.085064
H	5.335022	7.477466	14.645216
C	5.995905	13.214774	17.365144
H	5.272094	13.973978	17.060763
C	6.162552	12.044763	16.640762
H	5.573386	11.885667	15.734901
C	2.065616	9.967604	19.633770
H	1.141731	10.537169	19.506634
C	4.455259	8.526495	20.009137
C	3.282029	10.500651	19.205425
H	3.316576	11.488531	18.738643
C	4.463115	9.785385	19.391972
H	5.412452	10.212500	19.057113
C	2.042504	8.717880	20.250230
H	1.100064	8.295642	20.608452
C	3.227894	8.005107	20.434554
H	3.195370	7.024380	20.918181
C	6.174296	7.075674	18.859287
H	7.104906	6.504118	18.996934
H	5.406510	6.379051	18.490717
H	6.351530	7.822495	18.072087
C	5.732329	7.740601	20.160178
H	6.531782	8.412624	20.517372
H	5.596988	6.973174	20.940126

1@ST

142

E_e^{Sol}: -2960.82930783

N	12.842295	16.434050	16.314602
N	4.794749	4.899898	19.822731
N	2.220212	15.313305	24.330369
N	13.299121	13.938939	15.047332
N	3.768321	17.567662	23.496873
N	6.901205	4.656701	17.929867
C	13.829631	15.160976	14.510954
H	13.102427	15.663526	13.836366

C	9.512046	10.986207	15.536855
H	8.470793	10.772655	15.283447
C	2.457604	17.709543	24.068813
H	1.663321	17.635266	23.294251
C	10.125100	12.127894	15.031006
H	9.565747	12.796629	14.370419
C	8.090551	6.993445	16.673643
H	7.443922	6.364331	16.057907
C	12.645956	16.039630	17.504400
H	13.431609	15.503868	18.076491
C	3.821455	5.700212	19.680115
H	3.036096	5.532710	18.912643
C	10.204495	10.124587	16.400291
C	14.094384	16.137860	15.677591
H	14.798145	15.626325	16.370186
C	4.885135	3.752430	18.958614
H	4.333928	3.916077	18.007990
C	12.088427	13.638999	14.814063
H	11.442300	14.280895	14.180245
C	9.555926	8.915800	16.948704
C	8.920370	16.599303	19.562631
C	11.448862	12.436504	15.364201
C	10.024682	16.026498	20.211216
H	9.926620	15.687840	21.245410
C	6.364039	3.508226	18.609061
H	6.880199	3.339376	19.579422
C	9.129031	7.416843	18.808367
H	9.282453	7.149096	19.857647
C	11.229573	15.845283	19.540248
H	12.073941	15.379395	20.056646
C	5.162772	17.054802	21.603625
C	9.070639	16.994182	18.220831
H	8.229994	17.463499	17.704380
C	7.630473	16.768250	20.262029
C	2.342741	19.058539	24.777776
H	3.165862	19.132059	25.509507
H	2.509069	19.861045	24.041496
C	8.709800	8.120580	16.153456
H	8.558679	8.385075	15.104055
C	2.207130	16.552700	25.056483
H	3.014207	16.591464	25.820269
C	9.752194	8.544313	18.286525
H	10.374005	9.167523	18.933151
C	3.869734	17.228704	22.278147
H	2.973344	17.052505	21.648463
C	8.295379	6.624378	18.011184
C	6.418554	16.551780	19.590791
H	6.430537	16.229759	18.547459
C	11.371847	16.242946	18.205904
C	15.120209	14.883844	13.740881
H	14.895761	14.200693	12.906105
H	15.809745	14.341762	14.410908
C	10.273843	16.823943	17.554779
H	10.398779	17.137628	16.516135
C	3.393689	9.323578	21.945685
C	7.676641	5.421383	18.581810
H	7.941998	5.208170	19.637516
C	11.530376	10.448022	16.742161
H	12.090989	9.778495	17.399044
C	3.277028	10.611359	22.662553

C	0.998387	19.223152	25.474418
H	0.192820	19.243758	24.718437
H	0.958858	20.192725	25.996297
C	2.363555	12.863307	22.819652
H	1.729835	13.665220	22.435089
C	15.768330	16.167958	13.239105
H	16.707344	15.935926	12.711420
H	15.105075	16.647829	12.497015
C	5.204329	16.692375	20.252395
H	4.269896	16.505354	19.715681
C	7.582963	17.123560	21.622631
H	8.515303	17.316430	22.158949
C	16.023117	17.139940	14.385990
H	16.451081	18.082352	14.008160
H	16.777250	16.707320	15.067894
C	3.666228	6.914712	20.490580
C	2.331310	8.818445	21.179698
H	1.377830	9.350343	21.158487
C	4.725415	7.402232	21.269229
H	5.664125	6.845098	21.272614
C	0.854692	16.728747	25.751097
H	0.713539	15.901340	26.464961
H	0.062224	16.623260	24.990030
C	14.742871	17.422439	15.161948
H	14.936776	18.089968	16.016803
H	14.011088	17.942244	14.519657
C	6.514464	2.259082	17.743915
H	7.581776	2.108737	17.516217
H	6.013236	2.444956	16.778044
C	3.025585	14.397888	24.679896
H	3.717934	14.528025	25.537644
C	2.453026	11.641735	22.170940
H	1.898012	11.491294	21.242299
C	4.301558	2.517901	19.652705
H	3.239554	2.707410	19.878348
H	4.810767	2.393823	20.624367
C	12.144477	11.580186	16.230515
H	13.178585	11.828751	16.478876
C	2.470138	7.639659	20.454816
H	1.634132	7.272622	19.852326
C	5.921432	1.031588	18.424070
H	6.014851	0.151111	17.768324
H	6.501837	0.802707	19.335992
C	4.593504	8.588102	21.973707
H	5.453756	8.982477	22.518223
C	4.011216	10.861437	23.831624
H	4.644350	10.077103	24.250994
C	3.105346	13.106312	23.985042
C	6.371983	17.264931	22.283301
H	6.327771	17.555098	23.335317
C	4.462594	1.262898	18.803308
H	4.058152	0.390374	19.341239
H	3.860882	1.367673	17.882659
C	0.742895	18.078457	26.448416
H	-0.249946	18.179414	26.915518
H	1.480044	18.128875	27.269885
C	3.931488	12.089827	24.477649
H	4.514575	12.262475	25.386848
C	6.483789	11.099313	19.164610
C	6.251703	9.918157	18.323511

H	7.087849	9.648127	17.670905
C	5.159461	9.144288	18.293750
H	4.284133	9.329850	18.922394
H	5.118839	8.269665	17.639598
C	7.694817	11.800228	19.043691
H	8.434716	11.464214	18.311320
C	5.546980	11.559378	20.105587
H	4.595570	11.038145	20.218811
C	7.028188	13.345737	20.777389
H	7.232390	14.219099	21.400907
C	5.815700	12.663894	20.905805
H	5.071954	12.995295	21.635606
C	7.963667	12.913893	19.837628
H	8.907339	13.451990	19.716501
